# Review of Top-of-Canopy Sun-Induced Fluorescence (SIF) Studies from Ground, UAV, Airborne to Spaceborne Observations

**DOI:** 10.3390/s20041144

**Published:** 2020-02-19

**Authors:** Subhajit Bandopadhyay, Anshu Rastogi, Radosław Juszczak

**Affiliations:** Laboratory of Bioclimatology, Department of Ecology and Environmental Protection, Faculty of Environmental Engineering and Spatial Management, Poznan University of Life Sciences, 60-649 Poznan, Poland; anshu.rastogi@up.poznan.pl

**Keywords:** sun-induced fluorescence, remote sensing, ground observations, UAV, airborne observations, spaceborne observations

## Abstract

Remote sensing (RS) of sun-induced fluorescence (SIF) has emerged as a promising indicator of photosynthetic activity and related stress from the leaf to the ecosystem level. The implementation of modern RS technology on SIF is highly motivated by the direct link of SIF to the core of photosynthetic machinery. In the last few decades, a lot of studies have been conducted on SIF measurement techniques, retrieval algorithms, modeling, application, validation, and radiative transfer processes, incorporating different RS observations (i.e., ground, unmanned aerial vehicle (UAV), airborne, and spaceborne). These studies have made a significant contribution to the enrichment of SIF science over time. However, to realize the potential of SIF and to explore its full spectrum using different RS observations, a complete document of existing SIF studies is needed. Considering this gap, we have performed a detailed review of current SIF studies from the ground, UAV, airborne, and spaceborne observations. In this review, we have discussed the in-depth interpretation of each SIF study using four RS platforms. The limitations and challenges of SIF studies have also been discussed to motivate future research and subsequently overcome them. This detailed review of SIF studies will help, support, and inspire the researchers and application-based users to consider SIF science with confidence.

## 1. Introduction

Sun-induced fluorescence (SIF) is a novel remote sensing (RS) signal for monitoring global vegetation status, encompassing its structural and functional activity from the canopy to ecosystem levels [1,2]. SIF is important not only for the researchers and scientists dealing with plants and ecosystems, but also useful for those working in broader fields related to biophysics, biochemistry, and the physiology of plants [3]. The measurement of fluorescence is a sensitive, non-invasive, and relatively simple technique [4,5]. The plant molecules absorb solar energy in the form of photons, and on the absorption of photons, the molecules get into their excited state. The highly energetic excited molecules release energy through vibration relaxation and photon emission, which is called fluorescence [3]. SIF originates from the initial reactions in Photosystem (PS) II and occurs at the wavelengths in between 650 and 780 nm, with the first peak at 690 nm (SIF_690_), whereas PS I fluorescence occurs in far-red/near-infrared spectrum (>700 nm) with a peak at 760 nm (SIF_760_). The full stretch of the SIF spectrum covers the wavelengths ranging between visible (VIS) to near-infrared (NIR) spectrum of 640–800 nm. Both PS II and PS I operate in a sequence and are commonly measured by two peak signals [6] identified by their usual wavelength positions at SIF_690_ and SIF_760_ for PS II and PS I, respectively [7]. However, several abiotic and biotic stress factors impact the photosynthetic response and trigger the dynamic regulation of the two photosystems. Between the two, the response of PS II is more active and dynamic, which ultimately influences the emission of SIF signals. The implementation of modern RS technology on SIF over terrestrial vegetation is highly motivated by the direct link of SIF to plant photosynthetic activity. The advancement of passive RS technology and the development of various prototypes, including commercial ground-based, airborne, and spaceborne sensors dedicated to SIF estimation, help scientists for a detailed understanding of plant functional activity. The monitoring of plant status and functional activity over different ecosystems using different RS platforms is currently a growing interest in the scientific community.

The state-of-the-art of optical and hyperspectral RS is dependent solely on the reflectance spectra obtained from different RS platforms. Remote sensing and plant scientists are using these reflectance spectra as a key component in the development of several vegetation indices [8] related to plant functional activity, modeling of potential photosynthetic rate, carbon budget estimation, etc. Such indices (e.g., Normalized Difference Vegetation Index, NDVI; Enhanced Vegetation Index, EVI; Photochemical Reflectance Index, PRI; Water Band Index, WBI) are providing information about plant status and reflecting structural, biophysical, and biochemical properties of plants such as chlorophyll content (Chl) [9], leaf area index (LAI), greenness [10], zeaxanthin content [11], water content [12], plant biomass [13], and many more. However, detecting plant activity using RS technology is still a challenge in terms of two major aspects: (1) the dynamic nature of the photosynthetic activity and (2) fluctuations in reflectance spectra obtained from different levels of RS platforms. Among RS signals, SIF is the only medium that can capture the dynamic nature of plant photosynthetic activity from ground-based, UAV, airborne, and spaceborne platforms. SIF is a direct strategy to capture and diagnose the actual vegetation functional status, as SIF is emitted from the core of photosynthetic machinery [1,14]. SIF is capable of estimating and detecting more accurate carbon assimilation rates and earlier stress symptoms rather than normal reflectance spectra and vegetation indices [1,15]. The first attempt to quantify fluorescence passively (i.e., without any artificial excitation source) was done in the 1970s [16,17], after which the research on SIF intensified. 

However, to realize the potential of SIF and to explore its full spectrum using different RS observations, a complete document of existing SIF studies is needed [18]. In the past, several SIF-related review articles have been published, among which Meroni et al. [1] and Mohammed at el. [19] attracted wide attention. Meroni et al. [1] reviewed more than 40 scientific papers (until 2009) focused mainly on the issues related to SIF estimation through RS observations. The authors grouped the scattered information about SIF estimations and addressed the major differences in measuring approaches, instruments, and experimental setups. The study was mainly focused on the RS data requirements (i.e., radiance or reflectance, multispectral or hyperspectral) for SIF estimation and different SIF retrieval techniques. The theoretical descriptions, advantages, and drawbacks of each retrieval method were also addressed in the study. Maintaining a similar context with Meroni et al. [1], recently, Ni et al. [20] have reviewed the different SIF retrieval methods (e.g., Fraunhofer Line Depth (FLD), full-spectrum Spectral Fitting Method (SFM), Singular Vector Decomposition (SVD), etc.,). This study only focused on the description of the retrieval methods along with its mathematical equations. Furthermore, only SIF retrieval techniques from spaceborne data using such methods were covered. However, ground-based, airborne, and UAV-based studies, as well as their retrievals, were ignored by this review. 

Mohammed et al. [19] demonstrated the 50 years of progress in SIF studies related to historical and current developments in SIF spanning from the last several decades. Their research incorporates the heritage to contemporary understanding from traditional fluorescence science to the modern-day estimation of SIF through advanced methods and radiative transfer modeling (RTM). Furthermore, this study dealt with the description of SIF retrieval techniques and the techniques for field and airborne SIF sensing. The advancement of spaceborne observations to understand the process of photosynthesis and stress effects on plants were also addressed. Finally, the progress, challenges, and future directions of SIF studies were also discussed by Mohammed et al. [19]. However, the in-depth review of existing SIF studies (1975–2019) from the ground, UAV, airborne, and spaceborne sensors along with its retrieval methods, applied instrument/sensors, target areas, and the aim of that study in a broad manner have never been studied and described yet. In this article, we have performed a detailed review of existing SIF studies (1975–2019) based on the ground, UAV, airborne and spaceborne observations that fulfill the existing research gaps in SIF science. The aim of this article is to provide an up-to-date comprehensive review of the SIF studies from canopy to ecosystem under different observational scales. This review will identify the research gaps in the field of SIF science and also enrich our existing knowledge about the plant functional activity at different hierarchical scales through novel SIF signals. In our work, we have discussed the comprehensive interpretation of each SIF study using four important RS platforms (ground, UAV, airborne, spaceborne). In association with extending the existing SIF reviews, the limitations and challenges of SIF studies have also been discussed to motivate future research on SIF. This review will help us to identify the research gaps in the field of SIF science and also enrich our existing knowledge about the plant functional activity at different hierarchical scales through novel SIF signal. We assumed that this detailed review of SIF studies will further support and inspire the researchers and applied users to consider SIF science with confidence.

## 2. Understanding of Sun-Induced Fluorescence (SIF) through Remote Sensing (RS)

In recent days, the current state-of-the-art of SIF is highly relevant and purely attractive research interest in scientific communities. Global space agencies such as the European Space Agency (ESA) and the National Aeronautics and Space Administration (NASA) considered fluorescence missions (i.e., the Fluorescence Explorer (FLEX) mission by ESA to be launched in 2023, OCO-3 mission by NASA launched on 4 May 2019, OCO-2 mission by NASA launched on 2 July 2014) as one of their key projects to gain in-depth knowledge about terrestrial ecosystems and vegetation. In 2015, ESA selected the FLEX (Fluorescence Explorer) satellite mission under their Earth Explorer 8 program as one of their future potential missions, which will be operated in tandem with ESA Sentinel 3 [21].

The graphical representation of the SIF mechanism is clearly shown by Meroni et al. [1]. As SIF is emitted from the core of photosynthetic apertures, thus, it is possible to be detected by the passive remote sensing techniques using high-resolution spectrometers and the Fraunhofer Line Depth (FLD) principle applied on the red and far-red regions of the spectrum [1,14,22,23]. 

In remote sensing, the estimation of SIF from radiances is mainly recorded at the top-of-canopy (TOC) or the top-of-atmosphere (TOA). SIF is a weak signal that typically constitutes 1–5% of the reflected radiation in the RED and NIR regions [24]. Dedicated SIF extraction algorithms exploit regions of the atmospheric spectrum where the incident irradiance is strongly reduced due to the absorption in the Earth’s atmosphere [25,26]. Two SIF signal peaks are characterized by two telluric oxygen absorption features, namely O_2_A at 760.4 nm and O_2_B at 687.0 nm. To detect such narrow atmospheric absorption bands, spectrometers should contain fine (Full Width at Half Maximum (FWHM) of 1–5 nm) or ultrafine (FWHM < 1 nm) spectral resolution for any detectable sensors at any RS platform. In most of the published papers, far-red SIF signal was estimated by the FLD principle in the near-infrared region or exploiting telluric O_2_ bands. The far-red SIF (O_2_A at 760.4) was estimated from tower data [25,27,28,29], aircraft [22,30,31], and from satellite platforms [26,32,33,34] at different degrees of accuracy [35]. Few studies have also reported the estimation of both red (O_2_B at 687.0) and far-red SIF (O_2_A at 760.4) from the tower [27,36,37] and airborne data [38].

In this review, we will do a comprehensive study of the relevant researches that have obtained SIF signals from canopy to ecosystem using different RS platforms.

## 3. Development of SIF Retrieval Methods

The interest of the global remote sensing community to estimate accurate and precise SIF signal is increasing, which is evident by the development of advance SIF retrieval methods. The accurate estimation of SIF and its dynamics are necessary in order to understand the complex feedbacks and exchange interactions in the global terrestrial system [39]. The full fluorescence spectrum covers the wavelength range from 650 nm to 800 nm. However, most of the studies either consider the solar Fraunhofer lines—Fe (758.8 nm) and KI (770.1 nm)—or the two O_2_ absorption bands—O_2_B (687 nm) and O_2_A (760 nm)—due to their spectral proximity to the peaks of the chlorophyll SIF emission spectrum [14,39]. SIF represents a small fraction of solar radiance reflected by plants and measured through high-resolution spectrometers. Since errors can propagate at each step of retrieval along with inadequate measurement protocols, insufficient calibration of the sensors and challenges in retrieval methods have encouraged SIF researchers and scientists to develop modern SIF retrieval methods and advanced models [39]. 

Meroni et al. [1] categorized the SIF retrieval methods into two major groups: (1) radiance based and (2) reflectance based. Within the radiance-based methods, they sub-categorized the following: (A) multispectral, which includes Fraunhofer Line Depth (FLD), 3-band FLD (3FLD), and corrected FLD (cFLD); and (B) hyperspectral, which includes improved FLD (iFLD), extended FLD (eFLD), and the Spectral Fitting Method (SFM). Along with the dominance of radiance-based multispectral methods (i.e., FLD, 3FLD, and cFLD), radiance-based hyperspectral methods (i.e., iFLD, eFLD, SFM) also became quite popular over the time for the retrieval of the SIF signal. On the other hand, there are also other methods used for SIF retrievals such as the reflectance ratio, derivative indices, and infilling indices, which areconsidered under reflectance-based methods. 

Below in Table 1, we have summarized the main widely used SIF retrieval methods according to their advantages and disadvantages. However, apart from the mentioned SIF retrieval methods, several other and less common methods were developed to overcome the limitations of the existing methods. Such developing phase methods include the Differential Optical Absorption Spectroscopy (DOAS) method [40], which is a computational method based on non-linear least squares algorithm for the observation of strong Fraunhofer lines outside the O_2_A-band due to different light scattering properties [32]; the Simplified Radiative Transfer Method [41]; the radiative transfer model based on Principal Component Analysis (PCA) [34]; Fluorescence spectrum reconstruction (FSR) [42]; the full-spectrum Spectral Fitting Method (F-SFM) [43]; the SpecFit method [44]; and the aFSR method [45]. These methods are either in the developing phase or a limited number of research articles have been published that are not sufficient to review their advantages and disadvantages.

In this section of the methodological interpretation, we do not become judgmental and evaluate all the methods and finally prescribe the best SIF retrieval method. However, recent publications by Cendrero-Mateo et al. [39] and Ji et al. [46] suggested that the SFM approach applied to high-resolution spectra provided the most reliable SIF estimations with the lowest error (<6%). However, the question remains that SFM always needs a very high-resolution spectrometer (<1 nm) with a good signal-to-noise ratio (SNR) to estimate the most accurate SIF values. But, a very-high-resolution spectrometer is not always possible to manage for measurement.

## 4. Review of RS Platforms for SIF Estimation Over Different Ecosystems

**Figure 1:**
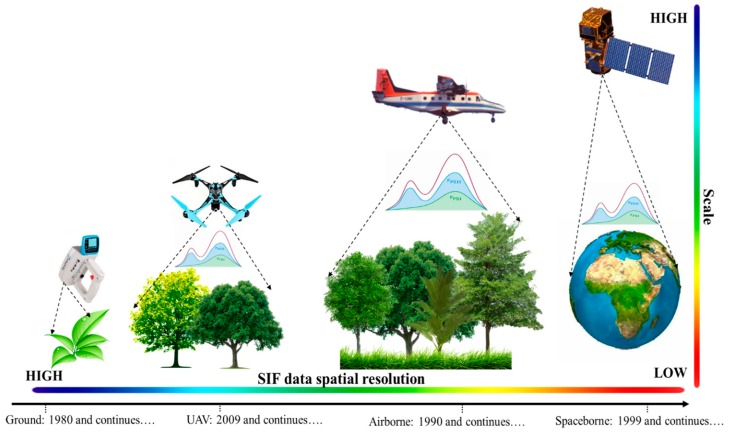
Different platforms used for top-of-canopy SIF measurments applied over time at different scales and spatial resolutions.

In this section, we will present a review of studies on fluorescence applications using ground-based, UAV, airborne, and spaceborne platforms from canopy to ecosystem level. This review will showcase the works from the pioneering work of the 1970s to present state-of-art knowledge. Associated Supplementary Tables (S1, S2, S3, S4) in various sections are showing the updated list of conducted studies at the ground, UAV, aircraft, and satellite platforms with the retrieval methods, instruments, target area, and aims of the work (after Meroni et al. [1]). A few years back, due to the unavailability of spaceborne and airborne missions, the majority of the research on plant fluorescence was performed at the ground level using ground-based instruments [1]. The early published works aimed to demonstrate the capacity of fluorescence signals to detect and track the plant stress and functional activity using ground-based instruments [1]. In 1931, the first observation of fluorescence over plants was discovered by Kautsky and Hirsch [7]. They observed that upon illumination condition imposed on a dark-adapted leaf, there was a rapid rise in fluorescence from PS II, followed by a slow decline, which is called the Kautsky Effect [62]. They concluded that the certain rise of fluorescence was in PS II, whereas PS I was constant. Based on the foundation of the ‘Kautsky Effect’, several spectrometers and sensors have been developed for fluorescence measurements from leaf to canopy levels. Such kinds of spectrometers (commercial, in-house developed instruments produced by companies and research institutes), airborne sensors (produced by research institutes, space agencies, and universities), and satellite sensors (produced mainly by space agencies) have been used nowadays to measure fluorescence signals with different spatial scales and resolutions (Figure 1).

### 4.1. Ground-Level Top-of-Canopy SIF Observations

Field spectroscopy has emerged as an important tool because of its various applications in different remote sensing applications. Field spectroscopy measurements are considered as a key scaling-up approach to understand the energy-matter interactions from leaf to canopy-scale studies [63]. The importance of ground spectrometers is that they are also necessary for the calibration and validation of airborne, UAV, and spaceborne sensors. The ground-based spectra are needed to be filled in numerous airborne and spaceborne sensor-based models for the purpose of estimating chlorophyll fluorescence [64]. The ground-based instruments are also capable of quantifying both incident and upwelling signals, which are important for fluorescence estimation in any retrieval method [1]. Due to its short optical path from the target (from centimeter to meter) to the sensor, the received spectra are not influenced by various atmospheric disturbances (e.g., dust particles, aerosols, water vapor, etc.). Thus, the atmospheric correction of measured fluxes is usually not performed in ground-based measurements [1]. For more details and a review of the physical and practical aspects of field spectroscopy measurements, see Milton et al. [64]. Both active and passive fluorescence techniques have become widely used over time with advanced improvements in measurement techniques and retrieval methods that developed the fluorescence science vividly. However, with the expansion of fluorescence science, scientists are keenly interested in understanding global terrestrial activity and photosynthesis through novel fluorescence signals. Currently, scientists are dedicated to solving issues related to the spatial, spectral, and temporal dynamics of vegetation fluorescence to deepen our ability to interpret large-scale vegetation functional activity. In this context, recent advancements of SIF measurement through passive sensors emerged as a promising technique to advance our knowledge in plant photosynthesis globally. We know that fluorescence estimation through active methods has a long history [19]. Many papers have been published on fluorescence measurements and their application based on active measurement techniques. Therefore, in this article, we have reviewed the passive sensor-based ground instruments used for top-of-canopy (TOC) SIF measurements and their associated studies over time. 

**Figure 2:**
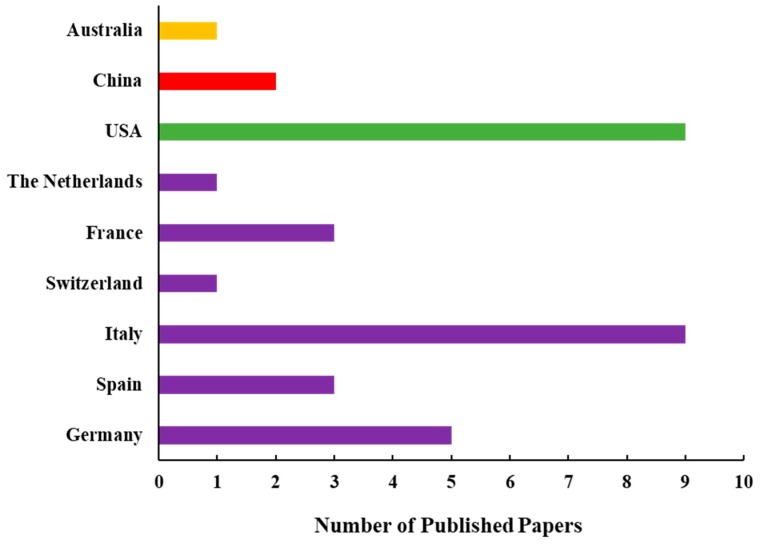
Bar graphs represent the country-wise ground-based SIF studies discussed in this review which were published until 2019. Color of the bar represents different continents (Yellow = Australia, Red = Asia, Green = North America, Violet = Europe). Note: the first author and/or corresponding author (if different) affiliations were used as the main criterion on defining the country of origin of the paper.

By analyzing the research papers published until 2019, one may see the domination of studies led by authors from Europe and the USA (Figure 2). Among 34 papers analyzed, 64% were published by European scientists, while 26% were published by American scientists. However, the number of SIF-related studies published by Chinese researchers is currently rapidly growing; hence, the global contribution of SIF-related studies will be soon distributed mostly among the three regions (Europe, USA, Asia).

Both in-house or laboratory-developed prototypes (e.g., FUSION spectrometer by NASA; USA, Multiplexer Radiometer Irradiometer or MRI by University of Milano-Bicocca; Italy, etc.) and commercial instruments (e.g., Fluorescence Box or FloX box by JB Hyperspectral, Germany; etc.) have been developed over time to measure SIF through passive measurement techniques (Table 1). Along with non-commercial prototypes, researchers have used commercial instruments for various approaches as they are very reliable from many perspectives, including the following. (1) The calibrated spectrometers provided SIF values in physical units. (2) Obtained spectra through spectrometers can easily be processed through very common and well-established methods for fluorescence estimation. (3) Most importantly, such commercial spectrometers blocked incident irradiance with short-pass filters (within the spectral window of SIF emission), which made the upwelling radiance with accurate SIF estimation [1]. Below, we have discussed several specific studies conducted through ground-based observations. These studies have represented the wide arena of ground-based observations from method and model development to the development of several new ground-based equipments at the current time to measure novel SIF signals. 

The detailed list of ground TOC-related SIF studies is provided in Table S1, while Table 2 summarizes the ground systems that have been used over time to estimate SIF. 

#### 4.1.1. Comparison between Active and Passive SIF Measurements

Passive SIF measuring instruments were used for several approaches from the comparison between active and passive SIF measurements, theoretical observation of SIF signals, plant stress detection to understanding the SIF-GPP (Gross Primary Productivity) relationship, and other purposes. Cendrero-Mateo et al. [65] compared active and passive methods of fluorescence measurements over wheat from leaf to canopy scales. A portable spectroradiometer (GER-1500, Geophysical & Environmental Research Corp., Millbrook, NY, USA) under the spectral range of 350 to 1050 nm with FWHM of 3.2 nm was used for passive SIF measurement, whereas Licor 6400 (Li-COR Biosciences, Lincoln, NE, USA) was used for active fluorescence measurements. The results concluded that at a single-leaf level, active and passive method-based fluorescence measurements were not comparable. However, canopy and leaf-average active measurements can be used to better understand the daily and seasonal behavior of SIF determined with passive measurements. Magney et al. [66] connected the active and passive fluorescence measurement by correlating the pulse-amplitude modulation (PAM) fluorescence parameters with spectra measured by QE Pro spectrometer (Ocean Optics, Douglas Avenue Dunedin, FL, USA USA). The authors observed a strong slop-dependent relationship between active and passive methods. Thus, the study is important for the purpose to show the SIF correlation with active fluorescence measurements, which can be further use to understand the plant physiology. 

#### 4.1.2. Method and Model-Based Studies to Estimate SIF from Ground Observations

In progress with the time, the estimation of SIF through passive methods were not only dependent on the instruments used for SIF measurements but also on various advanced algorithms (e.g., Singular Vector Decomposition (SVD), SFM, etc., see Table 1) and advanced radiative transfer models (RTM) (e.g., Soil Canopy Observation, Photochemistry and Energy (SCOPE), etc.,) developed to retrieve SIF signals from measured spectra. The development of such retrieval algorithms and models have further stimulated and reduced the knowledge gap between leaf to canopy scaling approaches. The iFLD method (which is the improved version of the well-established FLD method) was first proposed by Alonso et al. [54]. Mazzoni et al. [67] showed the preliminary results of the Extended Fraunhofer Line Discrimination (eFLD) method that exploits in the highest resolution (FWHM approximately 0.025 nm) until now found in the literature. The study was conducted over four attached single leaves of *Lycopersicon esculentum*, *Cucurbita pepo*, *Cucumis sativus*, and *Epipremnum aurea* plants using a double monochromator for SIF estimation. The study was enabled to disentangle the sharp difference in spectral windows for both oxygen absorption bands. In another study, Guanter et al. [68] applied a Singular Vector Decomposition (SVD) technique, which is a pure statistical algorithm that was applied over the paddy field in Italy to estimate fluorescence from reflected solar radiation. The outcomes have been compared with Global Greenhouse Gas Observation by Satellite–Fourier Transform Spectrometer (GOSAT-FTS) satellite data that showed a good agreement with O_2_A SIF retrievals. More challenging and difficult O_2_B fluorescence bands were retrieved by Mazzoni et al. [69] using a very high spectral resolution double monochromator (FWHM~0.025 nm) with the application of quadratic functions. Interestingly, without using wavelength and radiance-based instrument calibration, they received the O_2_B fluorescence signal in physical units. In this regard, Meroni et al. [1] suggested that the instrument calibration process should pass through the comparison of a measured raw incident irradiance spectrum with modeled values obtained from high-resolution Millimetre-wave Atmospheric-Retrieval Code (MARC) [70]. The radiative transfer model (RTM) based SIF estimation techniques were also quite popular over time in ground-based SIF studies. 

Among different SIF estimation techniques, the SCOPE model developed by ITC (University of Twente, The Netherlands) became popular over time. Van der Tol et al. [71] compared the measured and modeled estimation of diurnal and seasonal cycles of SIF over two croplands and two grass plots. The study used the SCOPE model to simulate SIF signals from portable spectrometers (HR4000, OceanOptics) and further compared them with tower data. The results showed that the simulated SIF signals matched with observed diurnal and seasonal cycles. Similarly, Liu et al. [47] used the ground reflectance spectra to obtain SIF values through the SCOPE model to understand the effects of spatial resolution and SNR effects on SIF retrievals. The model-based SIF values were further compared to spaceborne SIF values obtained from various satellites. The outcome showed that spaceborne SIF values were acquired with small to large errors (5–35%) in comparison to ground-based SIF values.

#### 4.1.3. Relation of Ground-Based SIF Measurements to Environmental Conditions

Photosynthetically active radiation (PAR) is the prime source of energy for plants to conduct fluorescence mechanisms. Passively measured SIF signals were tested under various environmental conditions, particularly under various PAR conditions in many studies. Moya et al. [72] studied a single bean leaf to understand the variations in fluorescence yield and reflectance in different sunlight conditions with changing PAR using a prototyped instrument (unnamed) based on the FLD principle. The study observed a positive correlation between the fluorescence yield and non-photochemical quenching (NPQ) processes at different PAR conditions. The same prototype was used by Moya et al. [29] to measure SIF from three types of green leaves and scaled up from leaf to canopy level under different PAR conditions along with gas exchange measurements. For leaf-level studies, they used a single bean leaf attached to a plant, and for canopy studies, they choose two different conditions: first, natural grassland in a controlled condition, and second, maize (*Zea mays* L.) crops in controlled stress conditions. A good agreement was found between SIF signals and gas exchange at the canopy level, whereas SIF signals were dynamic at the leaf level under different PAR conditions. In another study, Louis et al. [73] used a passive Multi-wavelength Fluorescence Detector (PMFD) sensor to measure SIF and reflectance on scots pine trees in the boreal forest. The study noticed that with the changing PAR condition, the SIF signal also changed. Liu et al. [74] showed a significant correlation between SIF_688_ and SIF_760_ bands with dynamic PAR conditions over winter wheat (*Triticum aestivum L*.) and Japan Creeper (*Parthenocissus tricuspidata*) plants.

#### 4.1.4. Application of Ground-Level SIF Observations for Plant Stress Detection

Apart from previously mentioned approaches, ground-based SIF observations were widely used for various application-oriented studies such as plant stress detection (natural or artificial). Early works by McFarlane et al. [75] used the ground-based FLD discriminator unit to the purpose of analyzing SIF from 13 mature lemon trees. The study found a significant relationship between plant water stress and SIF, where the irrigated and non-irrigated lemon trees were discriminated based on measured fluorescence signals using a 12 m high ground-based tower. 

In a subsequent work by Carter et al. [76], a Fraunhofer Line Radiometer (FLR) prototype instrument was used for the first time over palm and grape leaves exposed on DCMU ((3-3.4-dichlorophenyl)-1,1-dimethylurea) herbicide, which blocks electron transportation in PS II and enhances fluorescence emission [77]. The experiment showed that the enhanced SIF signal was coming from DCMU-treated palm trees, whereas no change in SIF emission was observed for control and treated grape leaves. The outcome revealed also the impact of stress condition through SIF signals along with the inverse relationship between fluorescence and the net CO_2_ assimilation rate. Evain et al. [78,79] also used the in-house developed prototype called passive multi-parameter sensor to understand the physiological modifications and SIF yield of maize plants in response to DCMU and water stress conditions. 

Kebabian et al. [80] developed a Plant Fluorescence Sensor (PFS) based on 688 nm and 762 nm-centered bands for SIF measurement using the FLD method. They applied the instrument for the single-plant canopy level measurement of greenhouse-grown bean plants (*Phaseolus vulgaris* L. var. Newport), which were fertilized with various levels of nitrogen (N) conditions. The outcome showed a statistically significant relationship between red and far-red SIF values with the degree of N treatments. The PFS prototype was further used by Freedman et al. [81] and Carter et al. [82] to understand the herbicide (Bromacil, a PS II herbicide) induced plant stress through SIF signal. Twelve Laurel oak (*Quercus hamispherica*) juveniles, corn, and soybean plants were selected for this study, where both of the studies received similar kinds of outcomes. Freedman et al. [81] found a higher ratio of SIF between F_687_/F_760_ in treated plots than in control plots, whereas Carter et al. [82] recorded an enhanced SIF signal at both O_2_ bands from treated plots. However, a strong negative correlation was also found between the SIF ratio and net CO_2_ exchange for Laurel oak leaves by Freedman et al. [81]. 

Meroni et al. [83] studied the relationship between fluorescence and reflectance at both oxygen absorption bands over control and DCMU-treated plots. The study used the SFM method to retrieve SIF signals. The high spectral resolution commercial spectrometer (HR2000, OceanOptics, USA) with the FWHM of approximately 0.2 nm was used, which facilitated the application of the SFM method. The outcome of the study showed that SFM allows estimation in a negative slope for the emission at 760 nm and also enabled the prediction of the wavelength where SIF reduced to 0, which is expected to occur at 787 nm [1]. However, this study did not imply the model in the O_2_B band, assuming that linearity may not be suitable for the red SIF region, which was in agreement with Gómez-Chova et al. [49].

Another study by Meroni and Colombo [51] analyzed the impact of DCMU treatment over SIF magnitude. The study was conducted over two potted beans (*Phaseolus vulgaris*) plants using HR2000 spectroradiometers (FWHM approximately 0.06 nm) under the spectral range of 635.5–802.5 nm to measure the SIF signal. The outcome revealed that the magnitude of the SIF signal of the DCMU-treated plot was four times greater than the control plant, indicating the damage of the photosynthetic apparatus of the bean plant due to artificial stress caused by DCMU treatment. 

In a separate study, Meroni et al. [84,85] measured optical signals from the leaf and canopy level in an ozone stress detection experiment over poplar clone (*Populus deltoids × P. maximowiczii*) and White clover plants (*Trifolium repens* L. cv. Regal) respectively using HR4000 spectrometers (OceanOptics, Douglas Avenue Dunedin, FL, USA) (FWHM approximately 0.13 nm). Plant physiological status with healthy and stressed symptoms were clearly discriminated through SIF signals from this experiment. 

Apart from the above-mentioned ground-based stress-related application studies, a review on the sensitivity of passive (sun-induced) and active (laser-induced) fluorescence due to water, temperature, and nitrogen-induced stress were covered by Ač et al. [86]. 

#### 4.1.5. Observation of Top-of Canopy SIF Daily and Seasonal Variations

SIF is highly dynamic in nature. Hence, the time-series analysis of SIF signals over different vegetation covers were conducted through different passively sensed instruments. Daumard et al. [87] calculated the SIF emission of a sorghum field during its growing period monitored by a TriFLEX passive fluorosensor (Table 2). The dynamics of fluorescence emission along with changing NDVI and PRI values were observed during the entire growing period. SIF was retrieved by the radiance-based filling-in method over 687 nm and 760 nm oxygen absorption bands. In the present time, Cogliati et al. [88] presented novel automated field spectroscopy (HR4000) constellated with SPEC_Full_ and SPEC_Fluo_ modules for collecting continuous and long-term SIF measurements. The second high-resolution spectrometer was dedicated to fluorescence retrieval based on the SFM method from sugarbeet, grassland, and lawn carpet canopies. This work was mainly designed for the implementation of the instrument for the continuous and seasonal monitoring of plant growth activity detection through SIF signals. Similarly, Rossini et al. [89] showed the variations of magnitude in emitted SIF signals over time in both oxygen absorption band regions from different plant species such as cropland, grassland, needleleaf forest, and deciduous broadleaf forest using three portable field spectrometers (two HR4000 and one QE65000, OceanOptics, Dunedin, FL, USA). The study implemented the SFM method for SIF retrieval that showed the highest SIF emission from crops followed by broadleaf and needleleaf species. In another study, Yang et al. [90] proposed a new instrument, FluoSpec2 (an automated system for collecting irradiance and canopy radiance), to measure the diurnal and seasonal variations of SIF from various ecosystems (Table 2). This paper mainly focuses on the instrumental design and calibration processes, and it provides future directions of the field spectroscopy system to measure SIF. The study used both the SFM and SVD methods to retrieve SIF signals over cropland and forest. 

#### 4.1.6. Application of SIF to Estimate the Gross Primary Productivity 

Furthermore, the SIF signal was also used for Gross Primary Productivity (GPP) fluxes estimation for different plant canopies. Such studies contributed evidence toward strengthening SIF-GPP and SIF-CO_2_ flux relationships and demonstrated the capability of the SIF signal to estimate gross photosynthesis rates and net CO_2_ fluxes from different platforms. In the earliest works, Carter et al. [91] measured the relationship between SIF and net CO_2_ assimilation rate over the leaves of three canopies (sweetgum, corn, loblolly pine). Moya et al. [29] also showed the relationship between SIF and gas exchange from leaf to canopy levels. Damm et al. [92] simulated the diurnal course of GPP of corn (*Zea mays*), winter wheat (*Triticum vulgare*), and beans (*Phaseilus vulgaris*) through SIF signals. Diurnal cycles of canopy radiometric response were captured by a high-resolution spectroradiometer FieldSpec Pro III (Analytical Spectral Devices, Boulder, CO, USA). The outcome showed that a significant improvement was noticed in predicting the GPP by including SIF into the modeling approach. In another study, Yang et al. [93] refined the SIF and GPP relationship at seasonal and diurnal scales over the temperate deciduous forest. A novel system (FluoSpec) was installed over the tower to measure SIF, whereas the eddy covariance tower-based measurements of CO_2_ fluxes were used for GPP estimation. Furthermore, this study was correlated with the SIF and GPP values with Global Ozone Monitoring Experiment-2 (GOME-2) and Moderate Resolution Imaging Spectroradiometer (MODIS) data, respectively. Similarly, Pérez-Priego et al. [58] used SIF and PRI to improve GPP estimations under varying nutrient level treatments in a typical Mediterranean savanna ecosystem. Migliavacca et al. [94] studied the mechanistic link between SIF and GPP along with the structural and functional controls of SIF_760_ under nitrogen–phosphorus (NP) treatment conditions. The study was conducted over the Mediterranean grassland ecosystem, whereas SIF_760_ was estimated through the SCOPE model. The outcome showed that nutrient implementation made changes in the abundance of plant forms and biochemistry of the canopy structure, which ultimately impacts the SIF_760_ and GPP relationship. A similar kind of work was conducted by Martini et al. [95] to show the SIF_760_ and GPP relationship under nitrogen (N) and phosphorus (P) treatment conditions over the Mediterranean grassland. In another study, Wohlfahrt et al. [96] investigated the SIF–GPP relationship under short-term intense heatwave conditions over the Mediterranean pine forest. SIF signals were measured from fluorescence box (FLOX box) (JB Hyperspectral Devices, Düsseldorf, Germany) and also simulated from the SCOPE model. The results showed that GPP decreased linearly during the time of the heatwave, whereas SIF declined slightly initially and then dropped dramatically during the peak condition of the heatwave. 

#### 4.1.7. Development of the New Ground-Based Passive Systems for Top-of Canopy SIF Measurements

Over time, with the increasing demand for SIF estimation, a number of different commercial spectroradiometers with different spectral resolutions have been developed for SIF estimation at the ground level. Such developments were reported by published studies that were mainly focused on the development, design, and authentication of such instruments, as well as for the calibration and validation of airborne and spaceborne sensors. In this regard, Julitta et al. [24] did a comparative analysis among four commercial spectroradiometers (i.e., two Ocean Optics, USA, HR4000s (HRNR and HRFR): 670–857 nm and 645–810 nm, respectively; Ocean Optics QE Pro: 645–810 nm; ASD FieldSpec Pro: 350–2500 nm) to retrieve SIF from the ground platform. Their study incorporated the (i) University of Milano Bicocca’s Multiplexer Radiometer (Milan, Italy), (ii) Jülich Research Center’s S-FLUO Box (Jülich, Germany), (iii) NASA Goddard’s FUSION (Greenbelt, MA, USA), and (iv) the CNRS TriFLEX (Paris, France), which potentially were considered to be used at the ground validation stations to support the future FLuorescence EXplorer (FLEX) Earth Explorer 8 Mission of ESA (for more details, refer to the following spaceborne-related section). The results showed that accurate far-red SIF signal estimation requires ultrafine resolution (less than 1 nm), whereas a higher spectral resolution (less than 0.5 nm) is required to accurately estimate red SIF signal. This study suggested also that SNR plays a crucial role in the precision of the far-red SIF measurements. Recently, Remote Sensing (ISSN 2072-4292) had published three manuscripts in its Special Issue “OPTIMISE: Innovative Optical Tools for Proximal Sensing of Ecophysiological Processes” addressing the review of Proximal Spectroradiometers for SIF estimation by Pacheco-Labrador et al. [97], Aasen et al. [98], and Cendrero-Mateo et al. [39]. First, Pacheco-Labrador et al. [97] discussed the effects of instrumental noise and biases on SIF retrieval. This review also highlights the associated uncertainties that are required for the calibration and characterization of the state-of-the-art SIF-oriented spectroradiometers. After analyzing the different sensor model-based SIF retrievals, the outcome found that SIF retrieval was highly influenced by instrumental noise and bias, whereas reflectance factors are barely modified. Furthermore, this study found a good correlation between SIF retrieval error and instrumental-induced biases, which indicated that precise instrumental characterization and calibration are highly necessary for the accurate estimation of SIF. In the second manuscript, Aasen et al. [98] reviewed different approaches (mainly the hierarchical changes in the sensing) to measure SIF from the leaf to canopy level incorporating only ground and airborne platforms. In this review, the authors mainly focused on the instrumental aspects, protocols, measurement setups, quality checks, and data processing techniques. The details of each section in instrumental aspects and quality measures were widely described. The atmospheric correction schemes and the process for the enrichment of SIF data quality were also discussed. However, this work was only limited to ground and airborne sensing. No such discussions were done on spaceborne and unmanned aerial vehicle (UAV)-based SIF sensing. The third manuscript by Cendrero-Mateo et al. [39] widely reviewed the SIF retrieval methods, emphasizing only the FLD-like methods (i.e., 3FLD, cFLD, iFLD) and SFM. This paper has a similar context to those of Meroni et al. [1] and Ni et al. [20], where SIF retrieval methods were mainly reviewed. The uncertainties and biases related to the SIF estimation through different retrieval approaches were widely discussed. Furthermore, they compared the accuracy and sensitivity analysis in SIF estimation between FLD and SFM methods. The outcome showed that the SFM method applied to high-resolution spectra provided the most reliable SIF estimation with a smaller error rate. 

Recently, several FloX boxes (JB Hyperspectral, Germany) have been acquired by the European Space Agency (ESA) and are currently being used around Europe for ground measurements. The purpose of these measurements is not only limited to SIF research; they also support the ongoing FLEX mission (for calibration and validation of the SIF datasets). FloX box is an instrument that provides continuous observation of SIF. The fluorescence box (FloX) measures fine spectra with the help of an advanced QE Pro spectrometer (FWHM = 0.31 nm) by Ocean Optics, Inc., USA. The technical details about FloX box are provided in Table 2. FloX box perfectly works at natural light conditions and is possible for permanent outdoor installations. FloX box constantly monitors the SIF through the comparison of sequential measurements of light reflected and emitted from canopies using a downward fiberoptics and solar irradiance using an upward-directed reference fiberoptics. The installed spectrometers are embedded in a temperature-controlled box that maintains a stable level of dark current of the spectrometers that maximizes the SNR with less acquisition time. With all the algorithms developed, FloX box can be considered as an easy to use instrument for SIF estimation.

Another instrument that needs mentioning is PICCOLO-DOPPIO, which is a prototype instrument developed by the University of Edinburgh, UK for SIF measurement. The technical details of this system are provided in Table 2. PICCOLO-DOPPIO is a lightweight, wirelessly controlled, autonomous, dual-field-of-view spectrometer system that measures fluorescence under natural light conditions with the same spectrometer (QE Pro by Ocean Optics, Inc., USA) as FloX box. In this system, a double-bifurcated fiber optic is used to transfer light from the fore optics to the spectrometers. The main advantage of this instrument is its lightweight, which makes it possible to install the system not only in outdoor conditions but also on UAVs [99]. PICCOLO-DOPPIO has its own graphical interface (GUI) to visualize and analyze the measured spectra. PICCOLO-DOPPIO also provides high SNR within the less data acquisition time and is capable of performing under changing environmental conditions. Some of the algorithms for the purpose of extracting SIF from measured values by PICCOLO-DOPPIO are still under development.

#### 4.1.8. Limitations of Ground-Based Top-of-Canopy SIF Observations 

The ground systems for SIF estimations are based on in-house grown prototypes developed within different research projects. Over time with the growing demand in SIF estimation, the in-house prototypes were developed for commercial purposes with more technical advancements. Each of the systems used capabilities of the commercially available spectrometers, but due to their technical limitations (i.e., low field-of-view (FOV), limited coverage, high prices) and methodological constrains (SIF cannot be directly measured with passive systems), the new retrieval algorithms were developed over time in order to estimate SIF. Increased demands of the research market required also further improvements of spectrometers with higher spectral resolution and lower SNR, which allowed estimating SIF with higher accuracy. Many studies have shown quite significant accuracies in SIF signals in both oxygen bands, which were further used for airborne or spaceborne data calibration and validation purposes. This development started to be even faster within the last few years through the investments of ESA and NASA into Earth Observations missions such as FLEX-EU and airborne platforms such as *HyPlant* (discussed in the next paragraph), which enhanced the development of high-resolution spectrometers and the research on the new type of measuring systems such as FloX and PICCOLO-DOPPIO. Currently, these systems start to be state-of-the standardized ground platforms for automated and/or manual SIF measurements of canopies, especially since FloX is already commercially available and offered by the JB Hyperspectral (Düsseldorf, Germany) company.

### 4.2. Airborne Top-of-Canopy SIF Observations

To understand the local and regional patterns of SIF signals from various ecosystems, airborne sensors are the most efficient platforms. Airborne sensors coupled with imaging spectrometers (in most cases) provide the spatial patterns of SIF acquired by images [1]. However, a few challenges are associated with remotely sensed airborne images in this passive measurement system. Firstly, atmospheric noises are associated with airborne images. The SIF signal captured by the airborne sensors is linked with the reflected signal emitted by the target along with the atmospheric path radiance. Therefore, the ratio between emitted and reflected radiation is higher and less evident in comparison to ground measurements [1]. Presently, sophisticated approaches dealing with atmospheric correction models using RTMs were performed to eliminate the atmospheric noises before considering the SIF signals for further analysis [1]. Secondly, the geometric precision of the airborne images is also a crucial factor before considering remote sensing data. The lower geometric accuracy of the images can create wrong and unrealistic data in comparison to the ground originality. Hence, the geometric calibration of the sensors is highly recommended before flying. Thirdly, the accurate and precise atmospheric and geometric corrections of the airborne, as well as the spaceborne images, are highly dependent on the codes and instrument calibration. It was suggested that accurate instrument calibration is highly recommended for precise information [1].

**Figure 3:**
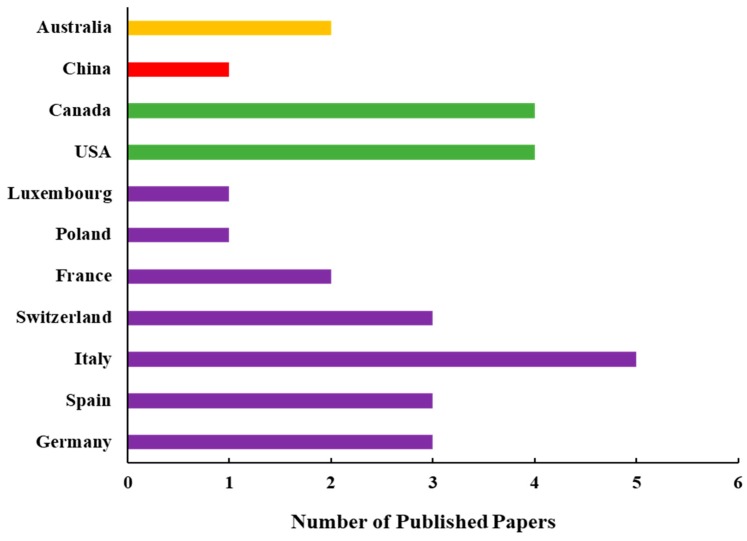
Bar graphs represent the country-wise airborne SIF studies published until 2019. The color of each bar represents different continents (Yellow = Australia, Red = Asia, Green = North America, Violet = Europe). Note: the first author and/or corresponding author (if different) affiliations were used as the main criterion in defining the country of origin of the paper.

According to our best knowledge, 29 papers were published to date that present the airborne-related SIF studies worldwide (Figure 3). Most (61%) of the papers were published by European researchers, and 28% were published by the North American scientists. The contribution of Asian and Australian studies is very minor and clear progress is not foreseen due to the lack of investment in suitable airborne apparatus in these regions of the world. 

The detailed list of airborne-related SIF observations are provided in Table S2, while Table 3 summarizes the airborne platforms used over time to estimate SIF along with their most important technical specifications. 

#### 4.2.1. First Airborne-Based SIF Study Using FLI

The first estimation of SIF based on airborne measurements was conducted by Gower et al. [100] over a marine ecosystem (Table S2). The aim of the study was mapping phytoplankton biomass using a Fluorescence Line Imager (FLI) imaging 8-channel spectrometer (spectral range 400–800 nm, FWHM = 2.5 nm, 1.0 km spatial resolution) mounted over aircraft operated by the Canadian Space Agency (Table 3). Over time, the importance of SIF measurement through airborne platforms has increased, which led to the development of the new imaging spectrometers with higher spatial and/or spectral resolutions. 

#### 4.2.2. ROSIS-Related SIF Studies 

Maier et al. [48] used the Reflective Optics System Imaging Spectrometer (ROSIS) operated by the German Aerospace Center (DLR) to understand and analyze the spatial pattern of SIF of crops at the Barrax study site in central Spain. The ROSIS was able to capture a surface-reflected radiance with a spatial resolution of 2 m and spectral resolution of 4.0 nm in the spectral range of 430–860 nm (Table 3). The study showed that the SIF signal gives different information from spectral vegetation indices, which provides additional insights into plant photosynthesis and plant stress conditions. The SIF retrieval process was based on the FLD method, whereas ground reflectance spectra were used to do the atmospheric correction of the ROSIS acquired images. In his previous study, Maier et al. [101] proposed a semi-empirical atmospheric correction scheme for retrieving the O_2_A SIF signal from non-fluorescing pixels, which were recognized as a patent. It was further used in his future studies.

#### 4.2.3. CASI-Related SIF Studies

Zarco-Tejada et al. [59,102,103] showed the variability of SIF signals through a hyperspectral Compact Airborne Spectrographic Imager (CASI, Itres Research Ltd., Canada) airborne sensor (spectral range of 380–1050 nm, FWHM = 2.5 nm, and spatial resolution from 0.5 to 2.0 m) (Table S2 and Table 3). Firstly, they investigated for the first time the relationship between airborne CASI hyperspectral canopy reflectance spectra and ground reflectance spectra with a Portable Chlorophyll Fluorometer (PAM-2000, Heinz Walz GmbH, Germany) through a Fluorescence–Reflectance–Transmittance (FRT) model over sugar maple forest (*Acer saccharum* M.) in the Algoma Region, Ontario (Canada) [59]. The study examined the ability of the airborne sensor to detect fluorescence and vegetation indices at leaf and canopy levels, which were finally validated through respective ground measurements. Furthermore, this study upscaled the leaf-level relationship between fluorescence and indices measurements to the canopy level through canopy radiative transfer models. Consistency was found between the leaf, laboratory and field canopy hyperspectral data. In subsequent work, Zarco-Tejada et al. [102] measured the effect of fluorescence on canopy reflectance signature under artificial and natural light conditions. The study was conducted over 12 sites of *Acer saccharum* M. in the same region of Canada. Similar to previous studies, ground-measured spectra, as well as airborne CASI data were used for analysis. The result showed that SIF was highly observed in the NIR zone, particularly in the red-edge spectral region. Zarco-Tejada et al. [103] showed the variability of SIF signals over maize growing under different nitrogen-induced conditions. The study used airborne CASI reflectance data to retrieve SIF based on the FLD method. The atmospheric correction scheme proposed by Maier et al. [101] was applied for SIF retrieval. The study showed a good agreement between CASI-derived canopy SIF values with the ground-truth leaf measurements of fluorescence. A CASI airborne sensor was further used to understand the water stress of the maize field (*Zea mays* L.) together with thermal AHS-160 (Sensytech Inc., Beverly, MA, USA) data by Rossini et al. [104]. Airborne SIF data and canopy temperature data showed a good agreement and demonstrated that SIF had high sensitivity in the low-temperature conditions during the afternoon. 

#### 4.2.4. ASIA-Related SIF Studies

In further progress, airborne SIF measurements were conducted by Corp et al. [105] and Middleton et al. [106] using an Airborne Imaging Spectrometer (AISA, SPECIM Spectral Imaging Ltd., Finland). The AISA captured a surface-reflected radiance in the spectral range of 450–900 nm with a spectral resolution of 1.56 nm and a pixel size of 2.5 m × 2.5 m (Table S2 and Table 3). The FLD method was applied for SIF retrieval. However, although the data were not corrected for atmospheric effects at both of the O_2_ bands, Corp et al. [105] was able to discriminate cornfields under different nitrogen treatments through a simple fluorescence ratio F_688_/F_760_ expressed in physical units. Middleton et al. [106] compared the SIF values calculated from ground-based ASD FieldSpec FR Pro (Analytical Spectral Devices (ASD) Inc., USA) and airborne AISA sensors over canopies of maize (*Zea mays* L.) under controlled nitrogen (N) conditions. The outcome showed that the fluorescence ratios (F_688_/F_760_) calculated from the airborne sensor were 10 times lower than the ground-based simulations because of the lack of atmospheric correction in the data. However, the ground observations were consistent with FluorMOD simulations [1,107,108]. Panigada et al. [30] used AISA airborne data to detect the water stress over maize (*Zea mays* L.) and sorghum (*Sorghum bicolor* L.) crops through SIF signal. The study used the FLD principle to extract SIF.

#### 4.2.5. AIRFLEX-Related SIF Studies

The first airborne multiwavelength passive sensor, AIRFLEX, was used by Moya et al. [50] over the succession of cultivated fields including alfalfa, sugar beet, and wheat under different phenological conditions at Barrax site, Spain (Table S2 and Table 3). AIRFLEX was a non-imaging (i.e., a targeting instrument) airborne sensor (FHWM < 1 nm, spatial resolution of 20 m) developed under the framework of the Earth Observation Preparatory Programme within the Sentinel-2 Fluorescence Experiment (SENT2FLEX) of ESA. The cFLD method was applied in this study to retrieve the SIF signals from airborne spectra. A second sensor, TERFLEX, which was identical to AIRFLEX, was used at the ground during the same time of flight to calibrate and validate the airborne data. This study conducted a sensitivity analysis to evaluate the effects of different flight altitudes (from 300 to 3000 m above ground level) on SIF band depth. In spite of different altitudes in flying, a significant outcome was achieved in both SIF bands, but a marginal signal degradation was found at 3000 m data in comparison with 300 m data. In a subsequent study, Daumard et al. [109] also examined the sensitivity of the O_2_B and O_2_A SIF band depths at varying altitudes, from 324 to 3123 m, on cultivated lands of Spain using the same airborne sensor. The study ultimately provides a correction model of altitude effects on the depth of O_2_ absorption bands.

#### 4.2.6. Non-Imaging Spectroradiometer-Related SIF Studies 

Further, Damm et al. [110] used FLD algorithms to retrieve SIF from a non-imaging spectroradiometer (ASD FieldSpec HR, Analytical Spectral Devices (ASD) Inc., USA) mounted in the low-flying research aircraft DIMONA (Metair AG, Menzingen, Switzerland) over a sugar beet (*Beta vulgaris* L. ‘Lucata’) field (Table S2). The system captured a surface-reflected radiance in the spectral range of 350–1050 nm with FHWM of 3.0 nm and the ground footprint size of 17–21 m in the long track and 1–5 m in the cross-track directions. The local sensitivity of SIF was investigated in this study and mostly focused on the atmospheric parameters to receive the accurate SIF values through the FLD algorithm. The results demonstrated that reliable and accurate SIF can be extracted through accurate knowledge of atmospheric scattering and absorption parameters. The total error budget of SIF was also estimated in this study. In a different work, Schickling et al. [111] demonstrated that SIF and the PRI index can act as a superior indicator for the actual efficiency of the photosynthetic machinery and can be used to estimate GPP as well. The study used ASD FieldSpec HR data measured from an ECO-Dimona aircraft (Metair AG, Menzingen, Switzerland) and ground data measured with the same spectroradiometer over a winter wheat field (*Triticum aestivum* L.) and sugar beet field (*Beta vulgaris* L.) in Germany. The 3FLD concept was used to retrieve the SIF signals. The outcome showed that SIF was more efficient at tracking the plant photosynthetic activity and GPP in comparison to spectral indices such as PRI.

#### 4.2.7. APEX-Related SIF Studies

The impact of varying irradiance on estimating vegetation indices (i.e., NDVI, PRI) and SIF was studied also by Damm et al. [112] using Airborne Prism EXperiment (APEX) imaging spectroscopic data (spectral range 400–2500 nm, FWHM 0.6–6.3 nm, spatial resolution from 2 to 5 m) (Table 3). The study was conducted over two types of trees, sugar beet and winter wheat canopies (Table S2). The FLD method was used to retrieve the SIF signals. The results showed the variation in vegetation indices and SIF estimations due to complex interactions of surface irradiance and reflectance anisotropy. In a different work, SIF_760_ and GPP relationships were studied by Damm et al. [113] over perennial grassland, cropland, and mixed temperate forest using APEX airborne imaging spectroscopic data. SIF_760_ was estimated from APEX-derived biophysical parameters through the SCOPE model, whereas GPP was calculated based on CO_2_ fluxes measured at the eddy covariance (EC) tower. The outcomes revealed that SIF_760_ and GPP relationship was asymptotic at the leaf level, whereas at the canopy level, it was more linear. 

#### 4.2.8. HyPlant-Related SIF Studies

In 2015, the first validated SIF images from HyPlant (airborne demonstrator for ESA FLEX mission) were published by Rascher et al. [14] showing the large spatial SIF variability of different vegetation types—i.e., trees, grass, sugar beet, corn, and potato—to showcase the potential variations in SIF signals over natural surfaces (Table S2). HyPlant is a novel airborne imaging spectrometer constellated with two modules i.e., (i) FLUO (670–780 nm, FHWM = 0.25 nm) used for SIF estimation and (ii) DUAL (370–2500 nm, FHWM of 3 nm in visible and near-infrared regions (VIS & NIR) and 10 nm in the short-wave infrared (SWIR)) for reflectance measurements (Table 3). The MODerate resolution atmospheric TRANsmission (MODTRAN)-5 [114] and 3FLD [48] method were used for atmospheric correction and the retrieval of SIF maps from the airborne data, respectively. The results showed a good agreement between airborne SIF maps with ground observations, and the spatial diversity of SIF was clearly detected at the regional scale. Recently, the technical aspects of a unique HyPlant sensor such as the spectrometer, its processing chain, details about the modules, and different processing steps for final data products were covered by Siegmann et al. [115].

Rossini et al. [35] provided the experimental evidence that links SIF at red and far-red regions with an actual photosynthetic efficiency. The experiment was conducted on controlled and herbicide-applied grass carpets using HyPlant airborne data. The outcome showed that the variations in the functional status of the actual photosynthesis rate were influenced by herbicide application. In a different study by Wieneke et al. [116], the GPP and stress conditions of different crops such as sugar beet, maize, rapeseed, and potatoes using HyPlant-derived SIF signals were evaluated. SIF was estimated through the iFLD method and validated by ground measurements. The results showed that along with estimating GPP, SIF had a large potential for tracking spatio-temporal plant adaptation in response to environmental stress conditions. In another study on the SIF–GPP relationship, Liu et al. [117] applied the downscaling of SIF from the canopy level to the photosystem level to better understand the SIF–GPP relationship over different species and different canopy structures. The study used SCOPE model-simulated SIF and HyPlant SIF data retrieved through the iFLD method to validate the random forest-based downscaling model outputs. Measurements were conducted over various species such as cotton (*Gossypium*) and different kinds of vegetables (i.e., sweet potato (*Ipomoea batatas*), Chinese cabbage (*Brassica rapa pekinensis*), thyme (*Thymus*), and pumpkin (*Cucurbita Cucurbita*) over three experimental sites of China. 

Based on SIF signals, the age of a managed loblolly pine (*Pinus taeda* L.) forest was estimated by Colombo et al. [118] using HyPlant and LiDAR data in North Carolina (USA). The Singular Vector Decomposition (SVD) method was implemented to retrieve red and far-red SIF from HyPlant data. The result showed that red SIF varied with forest stand age, whereas far-red SIF remained constant. A young pine trees stand exhibited nearly two-times higher red SIF than mature pine trees. The study concluded that photosynthetic stomatal conductivity may be a reason for such variations. In a different work, Tagliabue et al. [119] showed the relationship between GPP and absorbed photosynthetically active radiation (APAR) with both the oxygen absorption bands of SIF over a mixed forest site (Hardt Forest at Alsace) of France using HyPlant data. The outcome revealed that SIF_687_ has a non-significant relationship with GPP and APAR, whereas SIF_760_ has a significant non-linear relationship with GPP and APAR. The SIF maps from HyPlant data were derived through the SFM method. In subsequent work, Middleton et al. [120] combined SIF, reflectance, thermal information, and canopy structural information to understand the plant physiological process and forest ecosystem health of managed loblolly pine (*Pinus taeda* L.) forest using the same data. This study also used the SVD method to retrieve the SIF signal from HyPlant. The result showed ahigh sensitivity of the red fluorescence, indicating the responses were strongly affected by the diurnal temperature differences in the pine trees. 

Further, high-resolution airborne thermal infrared (TIR) together with SIF images were investigated by Gerhards et al. [121] to analyze the water stress symptoms of commercial grasses (*Festuca arundinacea* and *Poa pratense*) in Italy. The Telops Hyperspectral Thermal IR Camera (Hyper-Cam LW) and HyPlant SIF images were used in this study to investigate the sensitivity of the water stress over plots treated with anti-transpirant VaporGard (Miller Chemical & Fertilizer, Hanover, Pennsylvania 17331, USA) and kaolin. Surface temperature, the Crop Water Stress Index (CWSI), SIF indices (F_687_, F_780_), and vegetation indices were used as indicators in this study to understand the water stress. TIR-based indices showed a significant sensitivity on control and treatment plots during early morning and noontime. However, no significant SIF differences were observed due to water stress. In one of the recent HyPlant-related studies, Bandopadhyay et al. [122] examined the sensitivity of SIF and vegetation indices from various heterogeneous ecosystems (i.e., grassland, forest, and peatland) and over peatland plant communities (i.e., *Calamagrostietum neglectae*, *Sphagno recurvi-Eriophoretum angustifolii*, *Typhetum latifoliae*, *Cladietum marisci*, etc.) using HyPlant data. The degree of relationship between SIF and different vegetation indices were examined at a hierarchical scale (i.e., from the plant community level to ecosystem level) to demonstrate the big variability of remote sensing signals for different plant communities over peatland as well as different ecosystems. The SFM algorithm was applied to retrieve the SIF signals from HyPlant data. The study demonstrated for the first time the SIF (at 687 nm and 760 nm) maps for peatland and its surroundings. 

#### 4.2.9. Micro-hyperspectral Imaging Sensor-Related SIF Studies

The seasonal variability of SIF was observed by Zarco-Tejada et al. [123] using a micro-hyperspectral imaging sensor (spectral range 400–885 nm, FWHM < 7.0 nm, spatial resolution 0.4 m) mounted on Cessna aircraft over citrus (evergreen) crop (*Citrus sinensis* L. cv. Powell) under different water stress levels (Table S2 and Table 3). The study implemented a 3FLD method to retrieve the SIF signals to evaluate the potential of SIF to track photosynthetic activity at different phenological and stress stages throughout the season. The results showed a significant relationship between SIF and photosynthetic activity during stress and normal conditions. In subsequent work, Camino et al. [124] showed the effects of structural heterogeneity within tree crowns through airborne SIF data and the Crop Water Stress Index (CWSI). The micro-hyperspectral imager (Micro-Hyperspec VNIR, Headwall Photonics, Fitchburg, MA, USA) and a thermal infrared camera (FLIR SC655, FLIR Systems, Wilsonville, OR, USA) installed in tandem on board a Cessna aircraft were flown over an almond orchard field in Spain. The investigation showed a significant influence of canopy structure on both spectral angle change and SIF values. The same airborne hyperspectral imager was further used by Camino et al. [125] to understand the impact of nitrogen (N) concentration on wheat under irrigated and rainfed Mediterranean conditions through SIF data. The study was carried out during the 2015 and 2016 growing seasons over wheat fields. Results showed that SIF worked as a significant predictor of N concentration in plants under both conditions. In both studies by Camino et al. [124,125], SIF was retrieved through the FLD method.

#### 4.2.10. CFIS-Related SIF Studies

Alternatively to the above, Frankenberg et al. [126] demonstrated the technical aspects of the Chlorophyll Fluorescence Imaging Spectrometer (CFIS, Jet Propulsion Laboratory (JPL), NASA, USA) to map the far-red SIF from the aircraft (Table S2 and Table 3). The instrument was built for the purpose of evaluating SIF signals from Orbiting Carbon Observatory-2 (OCO-2) satellite data. The spatial resolution of the sensor was 30 m, in compatible to the Landsat sensor. The CFIS sensor can be able to differentiate and analyze the large-scale different crop types (corn, sorghum, soybeans, winter wheat, etc.). In the above-mentioned study, the FLD method was used to retrieve the SIF signals. The study demonstrated the mechanisms related to photosynthesis at fine spatial scales. 

#### 4.2.11. Limitations of Airborne Top-of-Canopy SIF Observations 

The deployment of high-performance airborne imaging spectrometers (i.e., HyPlant, APEX, CASI etc.) has opened the door to detecting SIF signals at local and regional scales. In contrast to ground-based SIF measurements, the airborne SIF measurements have been also evolved as a key component of the calibration and validation of the spaceborne sensors. However, the airborne platforms are used periodically and are campaign-based, aiming for testing the spectrometers and proving the research concepts, which farther are developed toward the new spaceborne platforms. Simultaneously, high spatial resolution, a good SNR, geometric precision, and wide spectral resolution are increasing the importance and value of airborne SIF measurements. However, low coverage area and high costs per campaign and huge data-processing costs (including time) are still remaining disadvantages of airborne SIF measurements. This is the main reason that airborne campaigns are not an affordable way to map the SIF signals for larger areas. Airborne SIF measurements are often carried out as one-time operations in comparison to continuous SIF monitoring through earth observation satellite missions. However, not many studies have been conducted through airborne SIF applications; still, more need to be done to better utilize and prove the big investments to the measuring systems by the ESA and NASA space agencies.

### 4.3. UAV-Based SIF Observations

**Figure 4:**
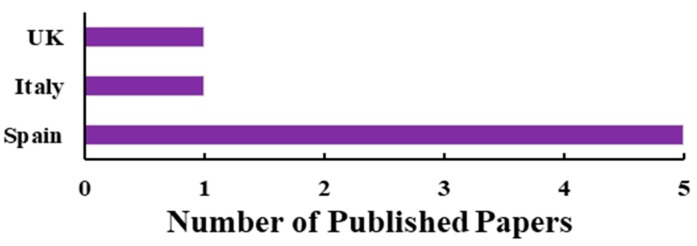
Bar graphs represent the country-wise unmanned aerial vehicle (UAV)-based SIF studies published until 2019. Violet represents the European continent. Note: the first author and/or corresponding author (if different) affiliations were used as the main criterion for defining the country of origin of the paper.

The big development of UAV-based SIF measurement is still missing in the existing literature. Based on our best knowledge, only seven research papers were published to date (all by European researchers) that present the SIF data acquired with UAV platforms (Figure 4). Similar to airborne observations, UAV measurements also need to be processed through the proper and accurate atmospheric and geometric correction process. UAV SIF measurements may be imaging data [31] or non-imaging data [127,128]. The imaging datasets are highly dependent on the configuration of the sensor, including spectral and spatial properties, whereas non-imaging data are mostly reliant on the spectral properties of the sensor. However, in both cases, validation of the SIF data is a crucial part before any further processing, because SIF signals are always highly sensitive to atmospheric properties. 

The detailed list of the UAV related SIF observations is provided in Table S3, while Table 4 summarizes the UAV platforms used over time to estimate SIF along with their most important technical specifications. 

The earliest work by Zarco-Tejada et al. [129] applied the in-filling method of SIF retrieval and fluorescence indices to detect the water stress of olive, peach, and orange orchards (Table S3). The study used the UAV mounted multi-spectral camera (MCA-6, Tetracam Inc., USA). The camera was operated within the spectral range of 400–800 nm and was equipped with bandpass filters providing two narrow-band channels (FWHM approximately 1.6 nm) centered at 757.5 and 760.5 nm particularly for the O_2_A absorption band (Table 4). The UAV-borne SIF maps were characterized with a high spatial resolution (15 cm pixel resolution) and validated by ground measurements using the Portable Chlorophyll Fluorometer PAM-2100 and Portable Gas Exchange Fluorescence System GFS-3000 (both of the same company, Heinz Walz GmbH, Germany). The results showed the high efficiency of the in-filling method to provide estimations of SIF from UAV images. SIF estimations were capable of demonstrating the water deficiency of plants similarly to derivative and reflectance-based indices. 

In subsequent work, Zarco-Tejada et al. [31] investigated the seasonal sensitivity of water stress level and stomatal conductance through SIF and PRI data from orchard trees using a micro-hyperspectral Hyperspec VNIR camera (Headwall Photonics, Fitchburg, MA, USA) on board a UAV (Table S3 and Table 4). The hyperspectral camera operated in the spectral range of 400–885 nm with 6.4 nm spectral resolution (FWHM) and 40 cm spatial resolution. The 3FLD method was used to retrieve SIF signals from hyperspectral images. Outcomes indicated that the water stress levels were significantly exposed by SIF signals. In another work, Zarco-Tejada et al. [130] showed the relationship between steady-state fluorescence and net photosynthesis, which was measured under natural light conditions at the leaf and canopy levels over non-irrigated vineyards. A UAV based micro-hyperspectral imager with previous configurations was used in this study, and the data were validated by ground measurements. The 3FLD method was used to retrieve the fluorescence signal from hyperspectral images. Outcomes showed a significant correlation between SIF and net photosynthesis measured at the ground targets. The study proved that through UAV-based high-resolution fluorescence imagery, it is possible to track the canopy photosynthesis process, but only in a stable atmospheric condition. Zarco-Tejada et al. [131] farther discussed the impact of spatial resolution on SIF retrieval from heterogeneous tree canopies of citrus orchards (Table S3). The Micro-Hyperspec VNIR camera (Headwall Photonics, Fitchburg, MA, USA) mounted on a UAV with the similar properties of the previous studies was used. The FLD method was used to retrieve the SIF signal from hyperspectral imagery. Due to canopy heterogeneity, the extracted SIF signal from aggregated pixels was degraded compared to pure tree canopies. A combined simulation model (FluorMOD + FLIM = FluorFLIM) was proposed in this study to extract the accurate fluorescence signal from heterogeneous canopy conditions. Using the same UAV-based system equipped with the hyperspectral imager (Micro-Hyperspec VNIR camera, Headwall Photonics, MA, USA), Calderón et al. [132] detected the disease infection (*Verticillium wilt (VW))* caused by the soil-borne fungus *(Verticillium dahliae Kleb)* on olive plants using fluorescence, temperature, and narrow-band spectral indices. The study used multispectral and hyperspectral imageries acquired through UAV platforms conducted during the spring and summer of 2009 to 2011. The FLD method was adopted to retrieve the fluorescence signals. The outcome showed that several vegetative indices and fluorescence signals had high potential for the early detection of *V. dahliae* infection and its discrimination. The study also concluded that fluorescence signals had a strong capacity to detect the infection at very early stages of disease development. 

In a different study, Garzonio et al. [127] discussed the technical aspects of using the small hyperspectral Unmanned Aircraft System (HyUAS) for measuring visible and near-infrared (VNIR) (VNIR) spectral reflectance and SIF signals (Table S3 and Table 4). The sensors were co-registered with high-resolution RGB cameras to support the calibration and validation of present and upcoming spaceborne and airborne products of Sentinel 2 and Sentinel 3. SIF and surface reflectance were measured with a high-resolution non-imaging spectrometer with a FWHM of 1.5 nm and spatial resolution from 0.5 to 12 m. Only far-red SIF was estimated in this study through the 3FLD method over the mixed forest, croplands, meadows, and some non-fluorescent targets such as bare soil. All the outcomes were further authenticated by respective ground validations. 

Very recently, a PICCOLO-DOPPIO hyperspectral non-imaging system was developed for the purpose of reflectance and SIF measurements as well as ground validation of airborne and spaceborne spectra [99]. The technical details about PICCOLO-DOPPIO were described in the ground part (Table 1). In a very early work, Mac Arthur et al. [99] reported the instrumental description, calibration procedures, and uncertainties related to the application of the PICCOLO-DOPPIO system for SIF measurements. The first attempts to install the PICCOLO-DOPPIO system on a UAV platform were made by Atherton et al. [133] (Table S3 and Table 4). They flew the PICCOLO-DOPPIO UAV system over a boreal forest site in Finland with the aim of collecting SIF measurements. However, no further processing and analysis of the collected spectra were reported in this paper apart from the instrumental description of the PICCOLO-DOPPIO system, flight plans, and sample sites. In another study, the PICCOLO-DOPPIO UAV system was used by Maseyk et al. [128] over the Free Air CO_2_ Enrichment (FACE) experiment in the UK. The UAV flights took place over a mature oak forest targeting on a developed treatment ring (typically in a ring arrangement) inside the forest. The SIF spectra were still to be processed (as reported in the paper), but initial analysis was shown to demonstrate the ability of the UAV platform equipped with the PICCOLO-DOPPIO system to measure reflectance and SIF from UAV. 

#### Limitations of UAV Based Top-of-Canopy SIF Observations 

Although not more studies have been published on UAV-based SIF measurements, over time, UAV-based SIF sensors have become popularized among scientists. The UAV-based SIF measurements can reduce the scale gap between temporally continuous ground measurements and spatially coarse satellite retrievals [133]. The general technical advancements of UAVs that allow for controlled deployment (e.g., low and slow flights allowing for high spatial resolutions and long integration times) strengthen the prospectives of UAV-based SIF measurements [19]. Furthermore, high spatial resolution, sufficient SNR, geometric precision, flexibility in data acquisition, and sensor integration have made UAV-based SIF measurements attractive. However, the uncertainties and error propagation associated with SIF data processing, due to the general lack of systematic analysis, and calibration errors are still a challenge in UAV SIF measurements. However, last year’s investments and SIF-related research enhanced by the ESA FLEX mission have been continually made toward the development of such UAV platforms to measure SIF at larger scales for the purpose of calibration and validation of upcoming satellites missions.

### 4.4. Spaceborne SIF Observations

**Figure 5:**
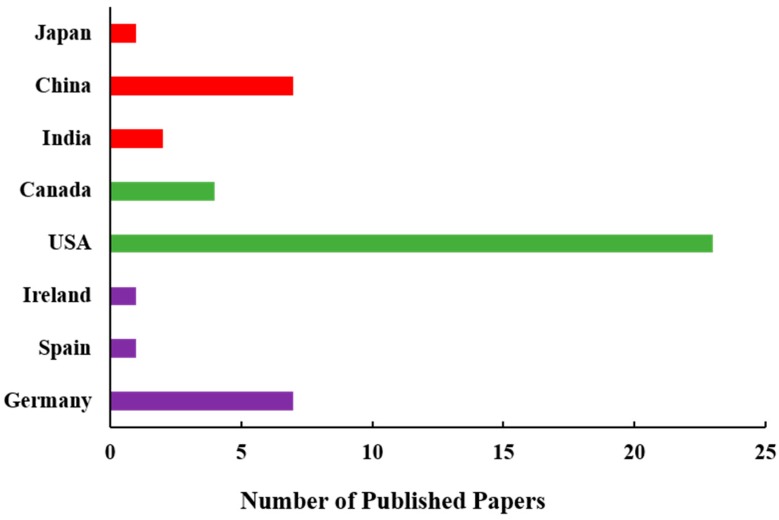
Bar graphs represent the country-wise spaceborne SIF studies published until 2019. Color of the bar represents different continents (Red = Asia, Green = North America, Violet = Europe). Note: the first author and/or corresponding author (if different) affiliations were used as the main criterion on defining the country of origin of the paper.

Enormous development has been noticed in the field of spaceborne SIF observations since Meroni et al. [1]. It is only possible due to some ongoing satellite missions dedicated to SIF and carbon measurements by global space agencies such as GOSAT (Global Greenhouse Gas Observation by Satellite) from JAXA, GOME-2 (Global Ozone Monitoring Experiment-2) from EUMETSAT and ESA, and OCO-2 (Orbiting Carbon Observatory-2) from NASA. Furthermore, the proposed FLuorescence EXplorer (FLEX) is a dedicated fluorescence mission operated by ESA that will measure SIF and reflectance from space to quantify global photosynthetic activity starting from 2023. Among 46 research papers published to date, the majority of them (58%) were published by North American researchers (Figure 5). Only 20% and 21% of published research studies were written by European and Asian researchers, respectively. 

The detailed list of spaceborne-related SIF observations is provided in Table S4, while Table 5 summarizes the spaceborne platforms used over time to estimate SIF along with their most important technical specifications. 

A few challenges are associated with spaceborne SIF measurements. Firstly, the SIF signal is very weak in comparison to reflected solar radiation, so the quantification of SIF through spaceborne sensors has so far not been feasible [134]. Thus, most of the remote sensing-based vegetation oriented studies are solely relying on spectral indices. Secondly, the SIF signal is very weak in long-wavelength and reflected radiations that crossed through a long atmospheric path until it reaches the receiver (sensor), so the influence of atmospheric challenges into the reflected radiation is always very high and sensitive. Similar to airborne and UAV measurements, a solid atmospheric correction is necessary before the further processing of spaceborne SIF data. However, all the standard data provided by the space agencies to the user community are generally atmospherically and geometrically corrected. Thirdly, the spatial resolution is always a big concern for spaceborne SIF estimations. As our global terrestrial systems are so heterogeneous in nature, the low spatial resolution of spaceborne SIF data (i.e., GOSAT: footprint size 10 km in diameter; OCO-2: footprint size is 1.29 km × 2.25 km, GOME-2: footprint size is 80 km × 40 km) is a limitation for local and regional level investigations (Table 5). Fourth, a major drawback of spaceborne SIF estimations is sensor sensitivity, which restricts imaging to relatively high concentrations under good solar illumination periods [135]. Simultaneously, the SNR, radiometric accuracy, and precise geopositional accuracy are common crucial points for spaceborne SIF investigations, which are also determining factors behind SIF data quality.

#### 4.4.1. First Spaceborne SIF Studies Using MERIS, MODIS

The very first spaceborne experiment conducted by Gower et al. [136] was aimed to retrieve SIF induced by ambient sunlight in order to detect and map the phytoplankton in Vancouver Island, Canada using Medium Resolution Imaging Spectrometer (MERIS) onboard the Envisat Earth Observation Satellite (ESA, Europe) (Table S4 and Table 5). Three spectral channels centered at 665 nm, 681.25 nm, and 705 nm were processed through the radiative transfer model based on the Fluorescence Line Height (FLH) algorithm to achieve the fluorescence signal. However, this study was only focused on the 685 nm fluorescence peak. The obtained fluorescence signals were further validated through airborne CASI-retrieved radiance spectra. This study was the first successful attempt that demonstrated, by combining FLH from MERIS red bands with chlorophyll estimations from MERIS blue and green bands, that it is possible to estimate the fluorescence ultra-weak signal from satellite data. In a subsequent work by Gower et al. [137], the FLH algorithm was applied to the data of MODIS onboard the Terra satellite (NASA, USA) to retrieve the chlorophyll fluorescence of phytoplankton. Here, the study used 665.5 nm, 677.6 nm, and 746.4 nm-centered bands to retrieve the fluorescence signals. Further, this study compared modeled fluorescence signals with MODIS fluorescence products and with sea-viewing wide field-of-view sensor (SeaWiFS) level 2 chlorophyll data that showed a good agreement between the modeled and original data. Gower et al. [135] also compared the potential of MODIS and MERIS data to retrieve the fluorescence peak near 685 nm using a similar method (Table S4). The outcome revealed that MERIS had more potential to achieve the fluorescence peak in comparison to MODIS due to its high SNR, flexible band placing opportunity, and the presence of a band at 709 nm in the baseline, which allows estimating fluorescence with higher accuracy. In this context, Grace et al. [138] mentioned that the fluorescence signal can be measured by spaceborne sensors for large spatial scales. However, very stringent atmospheric corrections—particularly aerosol optical thickness and terrain altitude [139,140] corrections—are required to use it effectively. On the other hand, they also added that it is challenging to perform highly accurate atmospheric correction over land surface in comparison to water due to its huge spatial heterogeneity. Simultaneously, the impact of atmospheric scattering on SIF retrieval was showed by Frankenberg et al. [141]. The study showed that fluorescence cannot be unambiguously discriminated from atmospheric scattering effects using O_2_ absorption lines. However, this study discussed only the FLD-based SIF retrieval technique from spaceborne sensors. Similarly, the impact of atmospheric influences on spaceborne SIF retrieval was also discussed by the subsequent study of Frankenberg et al. [142].

The next attempt by Guanter et al. [140] tested a new methodology to retrieve the SIF O_2_A signal from the images acquired from the MERIS spectrometer (Table S4 and Table 5). The study was conducted over the Barrax area of Spain, and they applied a new correction method (correction of atmospheric influences) based on the FLD principle or cFLD to MERIS bands 10 and 11 (centers at 753.8 nm and 760.6 nm; bandwidths = 7.5 nm and 3.75 nm, respectively) to retrieve the SIF signal. The SIF signal retrieval was processed through the Self-Contained Atmospheric Parameters (SCAPE-M) atmospheric correction scheme [143] and the validation of the signals was conducted through 3 m spatial resolution based airborne CASI images and ground measurements. Outcomes showed a good agreement between spaceborne SIF signals with airborne and ground measurements that demonstrate the feasibility of the spaceborne SIF estimations.

#### 4.4.2. GOSAT-Related SIF Studies 

Joiner et al. [41] made preliminary observations of global and seasonal trends of SIF from the GOSAT satellite (JAXA, Japan) data (Table S4 and Table 5). This study used the filling-in of the potassium (K) I solar Fraunhofer line method near 770 nm to derive SIF signals and SIF yield for the global vegetation. The MODIS EVI product was used for cross-validation and exhibited the different seasonality-wise variations in SIF and EVI. The results showed that the changes in the EVI lead to changes in the SIF signals for target regions. Frankenberg et al. [32] used GOSAT fluorescence data to understand the uncertainties and additional dependencies such as climatic factors in global GPP estimation. This study was conducted on aglobal scale, and it showed that a strong SIF–GPP relationship was found in boreal forests, whereas in other ecosystems such as savannas, croplands, and high-latitude needleleaf forests, the relationships were highly dynamic and uncertain according to seasonal variations. In a different study, global biome-dependent linear relationship between SIF and GPP was presented by Guanter et al. [33]. This study proposed a new method based on the modeling of the in-filling of solar Fraunhofer lines through fluorescence signals. The proposed method was a linear forward model derived by a SVD technique, which was able to perform a fast and robust inversion of top-of-atmosphere (TOA) radiance spectra to extract the SIF signals. The study was carried out on a global scale, and the potential of GOSAT-FTS data for global fluorescence retrievals was evaluated. The model derived fluorescence signals were further validated by ground-measured fluorescence signals. In a different study, Oshio et al. [144] identified the criteria for selecting vegetation-free areas to evaluate the zero-level offset through the comparison between derived SIF values obtained from GOSAT-FTS and OCO-2 data multiple spatial scales (footprint to global). The study was conducted over global ecosystems including non-vegetated areas such as bare soil. The results showed that no temporal variation of the zero-level offset was identified over a period of 9 years by using GOSAT-FTS and OCO-2 data. 

Similarly, Lee et al. [145] investigated the water stress impacts on plant productivity in terms of GPP using GOSAT SIF data over the Amazon forest region (Table S4). Lee et al. [146] simulated the SIF data from a new land surface model namely, Land Model version 4 (NCAR CLM4) developed by National Center for Atmospheric Research Community (NCAR), to estimate the terrestrial photosynthesis rate in terms of GPP, and further compared the simulated results with GOSAT SIF products. The study used the SCOPE model to estimate the SIF signals over global vegetation. Using GOSAT data on a global scale, they demonstrated that the simulated relationship between SIF and GPP values were close to accurate values. In a subsequent work by Parazoo et al. [147], the Dynamical Global Vegetation Model (DGVM) was applied to estimate GPP using GOSAT SIF data over the continents of North America, Europe, and tropical South America. The model-derived output showed the seasonal variability of GPP, which was highly varied for different global ecosystems such as tropical forests within the Amazon Basin, northern croplands, and deciduous forests. In this study, SIF was calculated through the average of two bands (757 nm and 771 nm) and two polarization techniques. Similarly, Parazoo et al. [148] investigated also the changes in seasonal carbon balance over southern Amazonia using GOSAT SIF data. The results showed the potential of GOSAT SIF data to estimate the carbon balance of biomes, which was very dynamic in nature during the wet and dry periods of the year. In order to increase accuracy of global SIF and GPP estimations based on GOSAT data, Köhler et al. [57] proposed a new fluorescence retrieval method called GARLiC (the GOSAT retrieval of chlorophyll fluorescence) to compare the Fourier Transform Spectrometer (FTS) method for GOSAT and two alternative methods (i.e., iterative least-squares fitting technique and SVD) proposed by Frankenberg et al. [32] and Guanter et al. [33]. The study used GOSAT data over global vegetation. 

#### 4.4.3. GOME-2-Related SIF Studies

However, considering the major limitations of GOSAT data with low spatial and temporal resolutions, GOME-2 (EUMETSAT, Europe) was considered attention in further SIF studies (Table S4 and Table 5). Using GOME-2 data, Joiner et al. [34] proposed a simplified radiative transfer model (fitting window algorithm) based on fluorescence retrieval techniques. This study provided a global coverage of SIF estimation with a spatial resolution of 0.5° × 0.5° within short days and revisit time. The proposed retrieval method was used for the mapping of far-red fluorescence with higher precision that was able to segregate the fluorescence and NDVI values on a global scale. A similar method was applied by Zhang et al. [149] to estimate the carboxylation (*V*_cmax_) in order to understand the photosynthetic capacity of corn in the Midwestern USA from GOME-2 data. Six flux tower sites located in the corn belt were further utilized for the physical validation of the experiments. The outcomes proved that space-based SIF data was a potential indicator to estimate the photosynthetic capacity of the crops. In subsequent work, Joiner et al. [150] applied a similar method on GOME-2 to track the seasonal cycle of photosynthesis. The values were further verified in relation to globally distributed diverse sets of tower gas exchange measurements for savannas, evergreen broadleaf, croplands, and mixed forests. The outcome showed the seasonal dependencies of photosynthesis in reference to space-borne fluorescence signals for all biomes. In a subsequent study by Köhler et al. [151], a linear method to retrieve the SIF signals was proposed to support SIF retrievals for medium spectral resolution instruments such as GOME-2 and SCanning Imaging Absorption SpectroMeter for Atmospheric CHartographY (SCIAMACHY). In this linear method, a backward elimination algorithm was applied that reduced the retrieval noise and automatically selected the number of state vector elements to optimize the coefficients of fit. In both studies, the outcome showed a good agreement with ground-measured SIF. In a similar context, Joiner et al. [152] proposed a new method—namely, stable solar Fraunhofer lines (SFLs)—to estimate SIF from GOME-2 and SCIAMACHY data. The study successfully mapped the reliable values of global monthly anomalies of SIF for the first time using thismethod. However, they applied this method only for the red SIF estimation rather than covering full SIF spectrum.

Guanter et al. [26] applied GOME-2 data to monitor crop photosynthesis in terms of GPP with fluorescence signals on a global scale. The study was conducted over USA croplands and European grasslands. The outcome of this study demonstrated that SIF-based crop GPP estimation was 50–70% higher and accurate than the state-of-the-art of existing carbon cycle models and vegetation products. Angular normalized values of GOME-2 SIF data were used by He et al. [153] to understand the SIF–GPP relationship under the sun and shaded conditions. The study was conducted over global vegetation, and SIF was retrieved through a fitting window algorithm in agreement with Joiner et al. [150]. The outcome showed that angular normalized values of SIF were better correlated with sunlit GPP in comparison to model-simulated GPP values. Furthermore, Wagle et al. [154] compared the potential of GOME-2 SIF data to estimate GPP with other model-derived outcomes such as the light use efficiency (LUE)-based vegetation photosynthesis model (VPM) and process-based SCOPE model over the maize (*Zea mays* L.) crop site in Mead, Nebraska, USA. The results concluded that the seasonal variation of GPP retrieved from SIF was accurately estimated compared to the model-derived outcomes. Similarly, the SIF–GPP relationship from the leaf to ecosystem level during the fall and spring seasons was examined by Yang et al. [155] using GOME-2 data. The study was conducted over a temperate deciduous forest at Harvard forest, USA. The overall result suggested that SIF can be a powerful tool to track photosynthetic rates at leaf, canopy, and ecosystem scales during seasonal variation.

In a different study, Guan et al. [156] monitored the crop yield and crop productivity using GOME-2 SIF data over the USA croplands. The outcome revealed that SIF provided the best measure of crop productivity in comparison to traditional crop monitoring approaches. Results were validated through county-level crop yield statistics. Similarly, Patel et al. [157] investigated the net primary productivity of croplands of the Indo-Gangetic plain using GOME-2 SIF data. However, this study used only raw SIF data from GOME-2 at 740 nm wavelength. 

The seasonal photosynthetic rates of continental evergreen boreal forests of mid to high latitude were examined by Walther et al. [158] using SIF and EVI from GOME-2 and MODIS data, respectively (Table S4). Further, they compared the SIF signals with green biomass content that showed a similar seasonality in deciduous forests. The study proposed that SIF was providing an unbiased detection of photosynthetic activity, even under seasonal variations. In subsequent work, Zhang et al. [159] compared the SIF–GPP relationship from a model-based perspective during the period between 2007 and 2012. The study used GOME-2 gridded temporal data and globally distributed 13 flux tower data (footprint size <1 km^2^) covering different biomes (i.e., cropland, grassland, evergreen needleleaf forest, deciduous broadleaf forest, and woody shrubland). This study mainly examined the dynamics of the SIF–GPP relationship from leaf to canopy scale on a short-term seasonal scale with the help of the SCOPE model. Yoshida et al. [160] investigated the relationship between SIF, NDVI, and the Fraction of Absorbed PAR radiation (FAPAR) during the 2010 Russian drought using GOME-2 SIF data and MODIS, NDVI, and FAPAR products. The outcome showed the declined trend in SIF as well as in NDVI and FAPAR caused by drought for all kinds of ecosystems such as mixed forest, croplands, and grasslands. Furthermore, the global land surface model-based estimated GPP from SIF data also declined significantly during the drought period over all the analyzed ecosystems. In this context, the monitoring of drought dynamics using GOME-2 SIF data was also described by Sun et al. [161]. The study was conducted over the great plains of the USA. Furthermore, the anomalies in SIF signals were calculated to represent the drought dynamics. Wang et al. [162] also evaluated the potential of SIF to monitor and assess droughts. SIF was found to be more sensitive in comparison to vegetation indices and short-term (one to two months) drought conditions and less sensitive to long-term (more than three months) drought conditions. The study was conducted over the great plains of the USA, and SIF was extracted through the FLD method from GOME-2 data. Similarly, the impact of heat stress on wheat production in the Indo-Gangetic region was investigated by Song et al. [163]. To understand the impact of heat stress on wheat productivity, they used GOME-2-derived SIF signals as well as NDVI and EVI indices. The results showed a 13.9% decline in SIF yield compared to the 1.2% and 0.4% changes in NDVI and EVI, respectively during the heat stress period. Large-scale variations in the northern high latitude forests’ phenology and the seasonal hysteresis of plant functions were captured by Jeong et al. [164] based on GOME-2 and GOSAT SIF data. The results showed that SIF was efficient to capture the hysteresis and plant phenology during the seasonal cycle. 

#### 4.4.4. OCO-2 Related SIF Studies

Along with GOSAT and GOME-2, the OCO-2 satellite (NASA, USA) was also used effectively for SIF studies and received major attention by the scientists (Table S4 and Table 5). In comparison to previous SIF detectable satellites such as GOSAT, GOME-2, the new OCO-2 SIF products have substantially improved the spatial resolution, data acquisition, and retrieval precision with higher accuracy [165]. Such valuable improvements in OCO-2 allow for the first time the validation of satellite SIF data against the ground and airborne measurements along with a more reliable estimation of terrestrial ecosystem functional dynamics in a spatio-temporal consortium [165,166]. The advancement of OCO-2 SIF data and its application to global photosynthesis observation was discussed by Sun et al. [165]. The technical overview of the OCO-2 SIF product, retrieval process, cross-mission comparison, and it’s potential to estimate GPP were delivered by Sun et al. [167]. The on-orbit performance of the OCO-2 satellite was discussed by Crisp et al. [168]. Frankenberg et al. [169] tested the OCO-2 SIF data in realistic conditions to evaluate the potential of OCO-2 for retrievals of SIF and its dependence on clouds and aerosols conditions. The study used the SVD method for SIF retrievals and the thermal vacuum test to verify the capacity of OCO-2 data. The study was carried out over global vegetation. An estimation of GPP from OCO-2 SIF data was demonstrated further by Li et al. [166]. The study was conducted over temperate forests of the USA, whereas the FLD method was used to retrieve SIF. The outcome showed a strong SIF–GPP relationship from OCO-2 SIF data validated by tower-based GPP estimates. In a similar way, Li et al. [170] investigated the biome-specific SIF–GPP relationship using OCO-2 SIF data. The study was conducted over different biomes of the globe (i.e., evergreen needleleaf forests, evergreen broadleaf forests, shrublands, and savannas), and the FLD method was used to estimate SIF from OCO-2 data. The results showed a strong agreement between SIF and GPP over different biome systems. Photosynthetic activity over the Arctic tundra region was investigated by Luus et al. [171] using OCO-2 and GOME-2 SIF data. The outcome showed that OCO-2 SIF data was more efficient to track the photosynthetic activity of the Tundra region in comparison to GOME-2 SIF data and MODIS EVI products. Verma et al. [172] discussed the influence of environmental conditions between spaceborne SIF data and tower-based GPP data over a growing season. The study used the OCO-2 data over the savanna grassland of Australia. The outcome showed that environmental factors played a crucial role in determining the nature of the relationship between SIF and GPP. Furthermore, the impact of bidirectional effects (BRDF) on the SIF–GPP relationship was discussed by Zhang et al. [173]. The study used OCO-2 data over different biomes such as mixed forest, woody savannas, and evergreen needleleaf forest. The results revealed that with different observational modes (i.e., Nadir, Glint, Target), the relation between SIF and GPP were influenced. In a different study, Smith et al. [174] tracked the seasonal to interannual GPP dynamics across the dryland ecosystem using GOME-2 and OCO-2 data. The study was conducted over different vegetation classes such as shrubland, savanna, and woody savanna, mixed forest, evergreen needleleaf forest, and grasslands located in New Mexico and the USA. The results demonstrated that the seasonal and interannual dynamics in GPP were better captured by SIF compare to EVI and PRI. Bacour et al. [175] examined the differences between OCO-2 and GOME-2 SIF products (mainly the differences in acquisition characteristics and processing chain) that impact the optimization of model parameters to estimate GPP. The study applied the data-driven principal component analysis method [164] to estimate SIF values. The study outcome showed that the potential SIF biases should be treated carefully in real-world SIF experiments in order to achieve realistic, accurate, and reliable future simulations. In a different study, Li et al. [176] developed high spatial and temporal resolutions (i.e., 0.05 degree, 8-day) SIF products (namely GOSIF) from OCO-2 and MODIS data. The study used a data-driven approach, particularly the Cubist regression tree model, to develop new SIF products for the period of 2000–2017. Newly developed GOSIF data showed the high correlation with tower-based GPP data on the global scale. 

#### 4.4.5. TROPOMI-Related SIF Studies

The potential of the upcoming TROPOspheric Monitoring Instrument (TROPOMI) on board the Sentinel-5 Precursor satellite mission has also received ongoing attention in SIF studies (Table S4 and Table 5). Improvements in SIF monitoring using TROPOMI data in comparison to GOME-2 were reported by Guanter et al. [177]. In this context, the first inter-sensor comparison between TROPOMI and OCO-2 SIF was conducted by Köhler et al. [178]. Finally, OCO-2 SIF was delivered as a benchmark in this comparison due to its higher spectral resolution, small ground pixels, and a lower standard error of across-swath SIF averages. However, TROPOMI SIF was considered for the purpose to fill the gaps left by OCO-2 in regions with spatial overlap. In a current study, Doughty et al. [179] showed an increase in the plant photosynthesis process in terms of SIF during the dry season. The study was conducted over the tropical Amazonian forest using TROPOMI SIF datasets. They retrieved the SIF signal from TROPOMI using a backward eliminating principal component method. 

#### 4.4.6. TanSat-Related SIF Studies

In very recent times, SIF was also retrieved from the Chinese TanSat satellite by Du et al. [180]. SIF was retrieved through the SVD method over global vegetation. The result showed a good agreement between SIF and MODIS NDVI, EVI, and GPP products. We hope that in the near future, more SIF studies will be published based on TanSat SIF products. 

#### 4.4.7. Other Optical and Hyperspectral Satellite-Related SIF Studies

Over time with the increasing interest in SIF studies, Gentine and Alemohammad [181] provided the machine learning approach to reconstruct SIF data from MODIS surface reflectance data in relation to original GOME-2 SIF products (Table S4). The outcome showed that the reconstructed SIF exhibits a much stronger seasonal and inter-annual correlation with tower GPP data than the original SIF data. Apart from the above-mentioned studies, there were few other studies where the technical aspects of SIF estimation were also investigated. Liu et al. [47] discussed the impact of spectral resolution and the SNR of the data to retrieve the SIF signals particularly from spaceborne sensors using FLD methods (i.e., FLD, 3FLD, iFLD). Experiments showed that the maximum error occurred from GOSAT (>35%) data, while the other sensors such as Thermal And Near-infrared Sensor for carbon Observations (TANSO)-FTS on GOSAT-2, SCIAMACHY, OCO-2, GOME-2, and FLEX FLORIS showed smaller error in between 5% and 20%. 

Besides the above, attempts were also taken to retrieve SIF signals from the Hyperion imaging spectrometer on board the Earth Observing-1 satellite (NASA, USA) (Table 5). Raychaudhuri [182] proposed a new method to extract SIF signals from Hyperion data over the vegetative areas of Kolkata city (Table S4). The proposed method compared the radiation ratio of the O_2_A band and separated the signals between vegetated and non-vegetated regions of the same image. However, in this study, no such atmospheric correction scheme was applied with the images, which would define the accuracy of the retrieved signals. In a similar study, Irteza and Nichol [183] applied the FLD method to extract the seasonal SIF signals of forest areas of Hong Kong from Hyperion data and compared the SIF signals with NDVI. No such good agreements were found between SIF and NDVI for all seasons, which might be the effect of inaccurate data processing. The authors claimed that water vapor and aerosols were not eliminated with great care from the Hyperion data and this was a reason for big bias in estimated SIF. In the coming years, the new satellites such as GeoCarb and Sentinel-4 are on their way to contribute significantly to future SIF studies. 

#### 4.4.8. Limitations of Spaceborne Top-of-Canopy SIF Observations 

A good number of scientific publications on SIF studies using spaceborne sensors have been reported over a time period of a few years [19]. The global space agencies such as ESA, NASA, and JAXA are continuously promoting and supporting the SIF science by their spaceborne SIF missions, which is evident by the OCO2 mission in 2014 by NASA and the upcoming FLEX mission in 2023 by ESA. The FLORIS satellite under the ESA FLEX mission is the first unique mission that was explicitly designed to deeply monitor and understand the photosynthetic activity of the terrestrial vegetation using novel SIF signals [21]. This mission will open new possibilities to assess the dynamics of the SIF signal, which will provide more detail about the core of photosynthetic activity in comparison to passive reflectance measurements by conventional land-surface monitoring satellites. The uniqueness of the FLEX FLORIS satellite is that the current observational satellites provide information about the amount of light absorbed by the plants, whereas FLORIS will provide direct insight into how much absorbed energy the plants are using for the photosynthesis process. This is a fundamentally new kind of information that has not been previously provided by the existing space observations. Another advantage of the FLEX FLORIS satellite is its high spatial resolution, which is quite good in comparison to other SIF monitoring satellites. However, FLORIS will only cover a particular region (mainly Europe), rather than the whole globe. 

The good point is that existing and upcoming spaceborne SIF measuring satellites (e.g., GOME 2, GOSAT, OCO2 etc.,) are providing us the synoptic coverage of SIF signals at regional to global scales in a temporal manner. Such repetitive coverage of spaceborne SIF sensors on board the satellite has strengthened our knowledge on terrestrial photosynthetic activity through the novel SIF signal over time to time. However, as SIF is very dynamic in nature, the low temporal repeatability of spaceborne SIF satellites is a major weakness in spaceborne SIF studies. Poor resolution is another issue that is associated with traditional SIF monitoring satellites. These are the main reasons behind avoiding spaceborne SIF data for monitoring heterogeneous ecosystems. Furthermore, atmospheric influences, poor SNR, poor spectral properties, precision error, and associated uncertainties are remaining challenges for spaceborne SIF estimations. However, over time, the development of advanced algorithms for spatial downscaling, advanced atmospheric correction modules, and the enhancement of spatial and spectral properties have made it easier to interpret the spaceborne SIF data. Most importantly, strong atmospheric correction is also required in this process of the quantification of SIF from spaceborne sensors [20]. Another key challenge of using satellite-based SIF data is that satellite SIF products are new in RS science and have a relatively shorter amount of data (GOSAT since 2009, GOME-2 since 2007, OCO-2 since 2014) in comparison to traditional RS datasets (such as MODIS and LANDSAT). Therefore, it is difficult to interpret and analyze the multiyear trends of photosynthesis and CO_2_ fluxes at the global or regional scales. To overcome such issues, new OCO-2 SIF products with much smaller footprints (1.3 × 2.25 km) are playing an important role. OCO-2 SIF products with fine resolutions are aggregated to a coarse spatial and temporal resolutions to monitor the temporal photosynthetic activity especially for local, regional, and global scales due to its sparse global sampling strategy. Studies such as those of Zhang et al. [184], Li et al. [176], and Yu et al. [185] demonstrated a way to improve the resolution of OCO-2 and extend the OCO-2 SIF products back to 2000. These improved SIF products have a higher resolution (0.05°), longer data record (from 2000 to present), and are globally contiguous; they will have more flexible and wide applications such as estimating higher-resolution GPP at local, regional, and global levels compared to the original ones [186]. 

The research in SIF science has achieved much progress over time with the help of strong retrieval algorithms where SIF can be estimated from reflectance-based satellite sensors. Studies have provided evidence that SIF can be estimated through the satellites such as MODIS, MERIS, and Hyperion. We assume that in the coming future with the development of advanced SIF estimation methods, it will be possible to derive SIF from other reflectance-based satellites. 

## 5. Conclusion and Future Prospects

The science of SIF incorporating modern RS technology is a rapidly emerging front advancing the knowledge in terrestrial vegetation and the global carbon cycle. Such wide and dynamic application prospects with its emerging capabilities make SIF highly attractive for global research communities. However, quantifying and applying SIF through different RS observations is an appealing prospect, but it is also very challenging [1]. In this article, we have provided an in-depth review of existing SIF studies from the ground, UAV, airborne and spaceborne measurements. However, a rule of thumb or finest practice to address the best method, application, instrument, calibration-validation process, and modeling have not been proposed. Few challenges and future directions are indicated here to motivate future research on SIF with confidence. First, the need for the validation of SIF signals is highly required, as SIF cannot be measured independently from vegetative targets; it can only be retrieved through dedicated methods. Hence, without actual measurements, original SIF values and the accuracy of the applied method cannot be determined. Second, it is highly necessary to understand and troubleshoot the sources of uncertainties that are associated with the SIF estimation process. The accurate estimation of SIF values in physical units and its correct interpretation is essential in application, as SIF estimations are highly affected by atmospheric (i.e., aerosols and cloud cover) factors and may depend on the instrument/system configuration and stability. Thus, the estimation of SIF should be done carefully, as such challenges need to be minimized or erased. Third, in reality, the use of a particular SIF retrieval method is restricted by the instrument or sensor availability [1]. SIF data collection through RS observations should be at very high spectral resolution, which will allow the user for subsequent resampling for multispectral or hyperspectral algorithm-based SIF estimations. Fourth, the validation of SIF values is a key concern for the leaf to ecosystem-level SIF applications. Such a requirement is highly necessary when one is working with heterogeneous ecosystems. The chances of mixing signals are more prominent in heterogeneous systems in comparison to homogeneous systems. However, validation is a real challenge for sensors and instruments with limited spatial resolutions [19]. In this scenario, new-generation efficient instruments, high-resolution UAV and airborne sensors, or satellite demonstrators (such as HyPlant for the FLEX mission) are overcoming this issue in a timely manner. 

In this context, the rationale behind the FLEX mission proposal is to successfully implement a satellite mission that will provide accurate SIF retrieval at both O_2_A and O_2_B absorption bands. Such a mission will help to reduce the spatio-temporal uncertainties associated with SIF as well as strengthen the global vegetation and carbon estimation models. 

In this review, we have discussed the in-depth interpretation of the existing SIF studies from the ground, UAV, airborne, and spaceborne observations. We have demonstrated the applied methods for SIF retrieval, instruments/sensors, target areas, and the aim of the previous studies. Over time, it has been observed that the acceptance of SIF has been increased in comparison to traditional optical RS-based vegetation monitoring approaches. The performance of a novel SIF signal (evident from publications) is a current interest for researchers in comparison to traditional vegetation indices. The vegetation indices are indicators of greenness, biomass, and water content, while SIF is directly linked to the photosynthetic mechanism. Much work has been done to show the superiority of SIF over spectral vegetation indices; for example, the SIF has been shown to be a stronger proxy of GPP than EVI [174], and it was more sensitive to drought [162] or other stress factors [75,76,77,78,79,80,81,82,83,84,85]. The underlying mechanism behind the observation is that the SIF contains more physiological information than the spectral vegetation indices [93]. The goal of several studies was not only to demonstrate the capability for the SIF signal, but also to showcase the development of SIF retrieval methods and its application over different targets. It has been proven that the application of SIF ranging from the monitoring of plant photosynthesis, stress detection, and plant growth monitoring has been fruitfully employed along with traditional RS-based reflectance-based methods.

There is a strong possibility for the development of SIF science, particularly in the field of ground, UAV, airborne, and spaceborne SIF studies. However, it is highly necessary to continue the future efforts toward the development of technological suits and operations to capture the reliable SIF signals in a spatio-temporal framework. Hand-held devices, mobile field instruments for ground measurements, advanced UAV sensors, advanced and precise airborne sensors, and spaceborne sensors with high SNR and radiometric stability can make a strong contribution toward the enhancement for future SIF science [19]. The efforts toward the development of airborne demonstraters for satellite missions (HyPlant for FLEX mission, CFIS for OCO-2 mission) along with the calibration–validation through novel ground systems (such as FloX box and PICCOLO-DOPPIO) are highly necessary in future SIF science to obtain accurate, reliable, and precise SIF values from any kind of homogeneous or heterogeneous ecosystems. Apart from this, systematic and efficient technical planning for ground, UAV, airborne campaigns, and spaceborne missions should be an important consideration in future SIF measurements. As SIF is highly dynamic and very sensitive to the atmospheric influences, the effective subtraction of such external effects for accurate SIF estimation should be always a prime concern in any kind of measuring campaigns. The capabilities and efficiencies in terms of the accuracy of SIF retrieval algorithms and models must be enriched so that they can work perfectly over any kind of vegetative surfaces. To get such a robust algorithm and accurate model, continuous efforts must be performed. In this regard, it is highly relevant to test all four kinds of RS platforms measured data, to develop best SIF retrievals algorithms and models. It will help to understand the plant functionality at any spatial scale. These SIF retrieval algorithms and models must be tested over both simple (grassland) and complex (forest, peatland) ecosystems where plant structural and functional diversity is maximum. However, to achieve the high accuracy of the models with reliable SIF values, sufficient spatial and temporal resolutions, high SNR, and radiometric stability is needed for any kind of SIF measuring platforms to avoid processing errors and reduce biases in SIF estimations. In a very recent trend, the assimilation of SIF datasets obtained from all four platforms along with supporting ancillary data with machine learning and deep learning modules could enrich the future of SIF science. 

## Figures and Tables

**Table 1:** Summary of the sun-induced fluorescence (SIF) retrieval methods.

SIF Retrieval Method	Category	Year of Development	Advantages	Disadvantages	References
Fraunhofer Line Discrimination (FLD)	Radiance based: Multispectral	1975	1. FLD algorithm perfectly discriminates between fluorescence and non-fluorescence targets without considering the impact by the absorption and scattering effects caused by atmospheric gases and aerosols. 2. FLD method only requires incident solar radiance and target radiance. 3. FLD provides SIF values in physical units.	1. FLD assumption is weak on broad absorption bands. 2. Highly variable spectral behavior of both reflectance and SIF in a spectral window. 3. The impact of atmospheric effects are completely ignored in the FLD method, which propagates error in the processing chain. The estimation of SIF is not worthy without considering accurate atmospheric parameters.	[1,17,20,39,47]
3-band Fraunhofer Line Discrimination (3FLD)	Radiance based: Multispectral	2003	1. 3FLD considers the linear variation of reflectance and fluorescence over the absorption window, which is a good proxy for red-edge shoulder toward short wavelengths. 2. A new radiance band is introduced (single reference band (λout) is replaced by the average of two bands out of the absorption line, at shorter and longer wavelengths) to overcome the limitations of the FLD method. 3. 3FLD provides SIF values in physical units.	1. The consideration of wide spectral intervals in the 3FLD method causes a violation of the basic assumption of linearity of reflectance and fluorescence. 2. A wide spectral interval creates an inaccurate estimation of SIF, particularly for the O_2_B band.	[1,20,39,48]
Corrected Fraunhofer Line Discrimination (cFLD)	Radiance based: Multispectral	2006	1. As in the upgraded version of the 3FLD method, cFLD applied two correction factors (α×r and αF) in order to capture the variation of the reflectance and fluorescence inside and outside the absorption bands. 2. cFLD method using the actual values of reflectance and fluorescence obtained from the sensor. 3. cFLD provides SIF values in physical units.	1. cFLD cannot be used for different vegetation types as it considers the interpolation of the reflectance and assuming constant fluorescence in the absorption band. 2. One of the correction factors in cFLD (α×) is computed from the interpolation from the reflectance rather than considering the actual correction factor obtained from fluorescence-free reflectance.	[1,49,50]
Spectral Fitting Methods(SFM)	Radiance based: Hyperspectral	2006	1. SFM replaced the FLD-like assumptions by using a spectral curve-fitting method to determine reflectance and fluorescence without using an apparent reflectance baseline. 2. SFM is a quasi-physical approach that processes two main components in a single platform: (i) atmospheric radiation transfer modeling; and (ii) decoupling reflectance and fluorescence in a single step without using apparent reflectance. 3. By using general mathematical functions, SFM makes use of all contiguous wavelengths to calculate reflectance and fluorescence within a specified spectral window highly focused around the two O_2_ absorption bands.	1. SFM requires very high spectral resolution spectrometers (e.g., Full Width at Half Maximum (FWHM) = 0.1 nm) to estimate gain and offset values. 2. SFM is restricted within a particular spectral range around two O_2_ absorption bands and not much focused on all contiguous wavelengths. 3. SFM requires complex setup and the overall computational time is very high.	[1,39,51,52]
Improved Fraunhofer Line Discrimination (iFLD)	Radiance based: Hyperspectral	2007	1. Considering the little peak in the reflectance spectrum due to fluorescence, iFLD computed one correction factor (α×r) used in cFLD by the cubic or spline interpolation technique that overcomes the assumption of linearity considered in cFLD and 3FLD. 2. iFLD removes atmospheric absorption effects with great care. 3. With the introduction of the hyperspectral radiances, iFLD provides SIF values in physical units. 4. iFLD was able to reduce the bias in fluorescence estimation, while FLD overestimates the fluorescence signal.	1. The actual correction factor obtained from fluorescence-free reflectance is not considered in this method; rather, it considers the interpolated version of the apparent reflectance 2. The outcome of the iFLD method is highly sensitive to the interpolation method, andthe best agreement was only obtained by using spline interpolation.	[1,53,54]
Extended Fraunhofer Line Discrimination (eFLD)	Radiance based: Hyperspectral	2007	1. With the help of very high spectral resolution (around 0.03 nm), in the eFLD process, it is possible to estimate fluorescence from three absorption bands. 2. In similarity with the assumption of the iFLD method, the eFLD method also removes atmospheric absorption effects with great care. 3. It provides fluorescence values in a physical unit.	1. This method only considers the variation in reflectance along with the variation in wavelength; however, eFLD does not consider the variation in the shape of the fluorescence spectrum along with the wavelength spectrum. 2. eFLD assumes that the local maxima in the incident irradiance spectrum have no absorption, which is not satisfactory in all conditions. 3. In a similar context to cFLD and iFLD, eFLD also considers the apparent reflectance instead of using actual reflectance.	[1,55]
Singular Vector Decomposition (SVD)	Radiance based: Statistical approach	2007	1. SVD provides fast and robust inversion of top-of-atmosphere radiance spectra that performs within two spectral microwindows (approximately 2–3 nm width) centered at 755 nm and 770 nm along holding many strong Fraunhofer lines. 2. SVD is a linear forward model that runs in an inverse way by non-weighted linear regression. It does not require the modeling of the instrument line shape function, spectral sampling, and the external solar irradiance dataset. 3. SVD is considering any radiance spectrum that can be linearly modeled as the combination of fluorescence-free and fluorescence components propagated to the top-of-atmosphere.	1. SVD considers the arbitrary selection of the optimum number of singular vectors that make an impact on retrieval accuracy and precision. 2. SVD is a statistical approach that opposes physical-based approaches. 3. A singular vector in the SVD method represents an average spectrum of the reflected light that is highly dependent on wavelength structures and instrument characterization.	[33,56,57]
(i) Reflectance ratio (RR) (ii) Derivative indices (DI) (iii) Infilling method or Infilling index (II)	Reflectance-based indices	Applied by Pérez Priego et al. [58], Zarco-Tejada et al. [59], Zarco-Tejada et al. [60], Dobrowski et al. [61]	1. The optical indices mainly exploit two or three narrow bands within the range of 650–800 nm (apparent reflectance spectrum in the red-edge region) to develop indices (or an index) that are related to fluorescence without implying any complex mathematical functions. 2. In considering two to three bands, one band should have fluorescence impact or should be located near the fluorescence peak, and the other should not be affected by the fluorescence. In this building process, the band normalization process is used to remove the influence of the reflectance shape. 3. Atmospheric correction is not necessary for the reflectance-based fluorescence measurement process.	1. The estimated fluorescence values derived from reflectance-based indices do not provide values in physical or ancillary units and are more prone to be impacted by plant chlorophyll content. 2. Reflectance-based methods to estimate fluorescence are mostly popular among laboratory-based experiments and have limited application in natural conditions, as such methods do not consider atmospheric influences in an open environment. 3. Reflectance indices estimating fluorescence are simply equivalent to vegetation indices, whereas fluorescence is a novel signal that emits from the core of the photosynthetic apparatus of plants.	[1,58,59,60,61]

**Table 2:** Characterization of the main instruments and measuring systems used for SIF measurements.

Instrument/Measuring Systems	Responsible Agency	Description of the Instrument/Measuring System	Field-of-View (FOV)	SIF Bands/Spectral Range (nm)	FWHM (nm)	References
Fraunhofer Line Discriminator (FLD)	The Perkin-Eimer Corporation Norwalk, CT, USA	Designed and patented for Perkin-Eimer Corporation. The instrument uses Fabry–Perot filters. The devices may be useful for any of the Fraunhofer lines, depending on the filter used, such as 486.1 nm (H-ß line), 589.0 nm (Na-D2 line), and 656.3 nm (H-α line).	37°	656.3	0.14	[16]
Fraunhofer Line Radiometer (FLR)	Earth Observation Research Office, NASA. Washington, D.C., USA	FLR is an electronic noise inherent radiometer and an advanced prototype that provides greater precision and accuracy in SIF measurements based on the FLD principle.	30° (approx.)	687	0.014	[76]
Plant Fluorescence Sensor (PFS)	Center for Materials Technology, Aerodyne Research, Inc., Billerica, Massachusetts 01821-3976. USA	Radiation reflected from plants passes through a low-pressure cell containing oxygen, where oxygen absorbs the energy and subsequently reemits photons, which are then detected by a photomultiplier tube.	28° (approx.)	690, 760	0.01	[80,81,82]
HR2000 high-resolution spectrometer	Ocean Optics Inc., Douglas Avenue Dunedin, FL, USA	A commercial spectrometer with a 2048-element Charged Coupled Device (CCD)-array detector for fluorescence detection.	25°	687–760 *	0.06	[51,83,84]
HR4000 high-resolution spectrometer	Ocean Optics Inc., Douglas Avenue Dunedin, FL, USA	A commercial spectrometer with a 3648-element CCD-array detector from Toshiba that enables precise optical resolution. The spectrometer is responsive from 200 to 1100 nm, but the specific range and resolution depend on the choice selected by the customer.	25°	690–800 *	0.13	[68,71,85,88]
QE Pro spectrometer	Ocean Optics Inc., Douglas Avenue Dunedin, FL, USA	A commercial spectrometer with very high sensitivity. Ideal for fluorescence measurements. It has high quantum efficiency and very stable cooled detector, SNR >1000:1. Spectral range is a customer-defined.	25°	640–800 *	0.31	[96,99]
Multi-channel spectrometer (MCS501)	ZEISS International, Germany	A commercial spectrometer that can be directly integrated into at-line, on-line, and in-line inspection processes. It gathers fast and precise results in fluorescence and layer thickness measurements as well as in plasma spectroscopy. The CCD or Photodiode Array Detector (PDA) sensors provide signal sensitivity in detecting low photon levels.	25° (approx.)	656, 762	0.3	[48]
Analytical Spectral Devices (ASD) FieldSpec	ASD-FR FieldSpec Pro, Analytical Spectral Devices, Inc., Boulder, CO, USA	The commercial spectroradiometer is a compact, field-portable instrument with a spectral range of 350–2500 nm. The fluorescence band might be estimated though FLD or based on other retrieval methods.	25°	685–760*	3.0	[54,74,92]
Passive Multi-wavelength Fluorescence Detector (PMFD)	The French National Centre for Scientific Research—(CNRS), Paris-Sud University, Orsay, France.	The PMFD instrument is a prototype built on a filters-based mechanism. Two filters are devoted to the measurement of the oxygen A band, and another two filters are devoted to the oxygen B band. The Fraunhofer line principle was applied to analyze the fluorescence bands.	4°	687, 760	0.7 1.3	[73]
High resolution dispersion apparatus	Nello Carrara Institute of Applied Physics. Florence, Italy	This is a prototype that consists of a low-stray light double monochromator with an f-number of 7.8 that consists of 510 active elements.	12° (approx.)	685–760 *	30 (modeled FWHM at 5 nm)	[68,69]
***Measuring Systems***						
TriFLEX	IPSL Dynamic Meteorology Laboratory (LMD), CNRS, France.	A prototype sensor with three spectrometers, where two identical spectrometers (HR2000+, Ocean Optics Inc., Dudenin, FL, USA) were used simultaneously to get irradiance and sample radiance spectra in the chlorophyll emission bands (630–815 nm). A third spectrometer (Ocean Optics Inc., Dudenin, FL, USA) measures vegetation radiance over a large spectral range. The fluorescence was estimated by FLD or another retrieval algorithm.	25° (approx.)	630–815	0.5	[24,87]
FluoSpec	Brown University, Providence, Rhode Island, USA	FluoSpec is a prototype instrument with an HR2000+ spectrometer (Ocean Optics Inc., Dunedin, FL, USA). The spectrometer was designed to be connected with a fiber optic shutter (FOS-2×2-TTL, Ocean Optics, Inc. USA) with two ports, each of which was connected to a fiber optic.	25°	680–775	0.13	[93]
FluoSpec 2	University of Virginia, Charlottesville, USA	FluoSpec with a modification of spectrometer where HR2000+ is replaced with a QE Pro Spectrometer (Ocean Optics, Inc., Dunedin, FL, USA). The advantage is a better SNR due to temperature control. The spectrometer can be optimized at different wavelengths as per the need. The QE Pro has an internal shutter that closes when dark current measurements are needed.	25°	730–780 650–730	0.14 0.4	[90]
S-FLUO box	Jülich Research Center and JB Hyperspectral. Germany	This prototype was assembled with two HR4000s (HRNR and HRFR; Ocean Optics Inc., Dunedin, FL, USA) and designed for the high temporal frequency acquisition of continuous radiometric values. A commercial optical multiplexer (MPM-2000, Ocean Optics Inc., Dunedin, FL, USA) is provided to switch between the upwelling and downwelling channels.	25°	680–755	0.2	[24]
FLUORESCENCE BOX (FloX)	JB Hyperspectral Devices, Düsseldorf, Germany	A commercial system using a QE Pro spectrometer by Ocean Optics Inc., Dunedin, FL, USA for fluorescence measurement. The spectrometer is further boxed inside a thermally regulated field encloser that further improves the SNR. An in-built shutter is provided to switch between the upwelling and downwelling channels.	25°	648–808	0.31	[96]
PICCOLO-DOPPIO	University of Edinburgh, Scotland	The prototype system use the QE Pro spectrometer by Ocean Optics Inc., Dunedin, FL, USA. In the system, a double-bifurcated fiber optic is used to transfer light from the foreoptics to the spectrometers. The system uses another spectrometer, FLAME-T-VIS-NIR of Ocean Optics Inc., Dunedin, FL, USA (Spectral range 400–1000 nm) for the purpose of calculating vegetation indices.	25°	650–800	0.31	[99]
Multiplexer Radiometer Irradiometer (MRI)	Remote Sensing of Environmental Dynamics Lab., DISAT, University of Milan-Bicocca, Milan, Italy	MRI is a prototype consisting of two HR4000 (Ocean Optics Inc., Dunedin, FL, USA) spectrometers, namely SPEC_full_ (400–1000 nm) and SPEC_fluo_ (700–800 nm) with different resolutions. An optical multiplexer (MPM2000, Ocean Optics Inc., Dunedin, FL, USA) was used to switch between the spectrometers.	25°	760	0.1	[88]
FUSION	Goddard’s Space Flight Center, NASA, Greenbelt, MA. USA	FUSION prototype assembled with two spectrometers (1) Ocean Optics USB 4000 Spectrometers (345–1040 nm) and (2) Ocean Optics HR 4000 Spectrometers (650–840 nm) with upward and downward viewing properties. FUSION also contains a CFmicro SF15 infrared sensor (8 to 14 µm).	25°	650–760	< 0.13	[24]

* *user specified*.

**Table 3:** Summary of the airborne platforms and systems used for SIF estimations until 2019.

Airborne Sensor	Responsible Agency	Filed-of-View (FOV)	Aircraft Carriers	Spectral Range (nm)	Spatial Resolution (m)	Number of Spectral Channels	SIF Bands (nm)	FWHM (nm)	References
Fluorescence Line Imager (FLI)	Canadian Space Agency, Canada	70°	DC-3, Falcon Fan-jet, Cessna 402, Piper Navajo, Dornier DO-228, Twin Pioneer	400–800	1000.0	8	685, 760	2.5	[100]
Reflective Optics Imaging Spectrometer (ROSIS)	German Aerospace Center (DLR) and Astrium, Germany	± 8°	DLR Falcon Jet, Dornier D0228	430–860	2.0	28	665, 762	4.0	[48]
Compact Airborne Spectrographic Imager (CASI)	ITRES Research Ltd., Alberta and York University, Canada	23.6° and 39.3°	CASA-212-200, Cessna 208B	380–1050	0.5–2.0	288	689, 751.8	2.5	[59,102,103,104]
Airborne Imaging Spectrometer for Applications (AISA)	SPECIM Spectral Imaging Ltd., Finland NASA, USA	21°	FLIS, Cessna 402B	450–900	2.5	288	688, 760	1.56	[105,106]
AIRFLEX	LURE Photosynthesis and Remote Sensing team in Paris, France ESA, Europe	2°	Cessna C 208B Gran Caravan aircraft, CNR SENECA aircraft	300–800	20.0	6	687, 760	0.5–1.0	[50,109]
Non-imaging ASD FieldSpec HR mounted in aircraft	Metair AG, Switzerland	1°	DIMONA research aircraft	350–1050	17–21 m in the long track flight direction and 1–5 m perpendicular to it	700	760	3.0	[110,111]
Airborne Prism Experiment (APEX)	University of Zurich; Switzerland the Flemish Institute for Technological Research: VITO, Belgium ESA, Europe	28°	HALO, Dornier Do-228	400–2500	2.0–5.0	312	760	0.6–6.3	[112,113]
HyPlant	SPECIM Spectral Imaging Ltd. Finland Forschungszentrum Jülich, Germany and ESA, Europe	32.3°	Cessna Grand Caravan C208B, NASA’s UC-12B aircraft	370–2500	1.0	622	670, 780	0.25	[14,35,115,116,118,119,120,121,122]
Micro-hyperspectral imaging sensor	Headwall Photonics, Fitchburg, MA, USA	50°	Cessna aircraft	400–885	0.4	260	760	5.0–7.0	[123,124]
Chlorophyll Fluorescence Imaging Spectrometer (CFIS)	Jet Propulsion Laboratory (JPL), NASA, USA	11.46 °	Twin Otter (DHC-6)	737–772	30.0	256	760	<0.1	[126]

**Table 4:** Summary of the UAV platforms and systems used for SIF-related studies until 2019.

UAV Sensor	Producer	Field-of-View (FOV)	UAV Carriers	Spectral Range (nm)	Spatial Resolution (m)	Number of Spectral Channels	Sensor Type	SIF Bands (nm)	FWHM (nm)	References
Multispectral camera (MCA-6)	Tetracam Inc. California, USA	42.8° × 34.7°	UAV helicopter	400–800	0.15	6	Multispectral imager with 6 independent image sensors	760	1.6	[129]
Micro-hyperspectral imager, (Hyperspec VNIR camera)	Headwall Photonics, MA, USA	50°	UAV consisted of a 5-m wingspan fixed-wing platform capable of carrying a 3 kg payload for 1.5 h endurance at 13.5 kg take-off weight (TOW) (Viewer, ELIMCO, Seville, Spain)	400–885	0.40	260	Hyperspectral imaging sensors	760	6.4	[31,130,131,132]
HyUAS: A small hyperspectral imager	Remote Sensing of Environmental Dynamics Laboratory. Department of Earth and Environmental Sciences, University of Milano-Bicocca, Milan, Italy	approximately 2.5° to 15°	UAV system developed by the Anteos platform (Aermatica S.p.A., Gironico, CO, Italy). UAV system provides a total take-off weight of 9 kg, and it is able to carry a maximum scientific payload of 2 kg for a total flight time of 20 min	350–1000	0.5–12.0	8	Hyperspectral Unmanned Aircraft System (HyUAS) assembled with a high-resolution spectrometer (USB4000 (Ocean Optics Inc., USA) with an RGB digital camera (12.1 Megapixel CMOS sensor, PowerShot S100 (Canon, Tokyo, JAPAN)	760	1.5	[127]
PICCOLO-DOPPIO non-imaging hyperspectral system	NERC Field Spectroscopy Facility, School of Geosciences, University of Edinburgh, UK	25°	DJI Matrice 600 Proquadcopter (DJI, Shenzhen, China)	400–1000	N/A	6	PICCOLO-DOPPIO assembled with high-resolution non-imaging spectrometer QE Pro and FLAME-S Ocean Optics Inc., USA	687, 760	0.31	[128,133]

**Table 5:** Summary of the satellite platforms and systems used for SIF estimations until 2019.

Sensor/Satellite	Status/Launch	Responsible Space Agency	Spatial Resolution/Footprint Size (km)	Temporal Repeatability	Spectral Range (nm)	Coverage	FWHM (SIF) (nm)	Signal-to-Noise Ratio (SNR)	References
Medium Resolution Imaging Spectrometer (MERIS)/Envisat-1 Earth Observation Satellite	Non-operational	ESA, Europe	Ocean: 1.04 × 1.20 Land and coast: 0.26 × 0.30	3 days	15 bands ranging in wavelengths 390–1040	Global	3.75 (around 753.8 nm used for SIF retrieval)	Good	[136,140]
Moderate Resolution Imaging Spectrometer (MODIS) / Terra and Aqua satellite	Operational	NASA, USA	MODIS acquires data at three spatial resolutions: 0.25 (bands 1–2), 0.50 (bands 3–7), and 1.00 (bands 8–36)	Daily, 4-Day, 8-Day, 16-Day	36 bands ranging in wavelengths 620–1390	Global	N/A	Poor	[135,137,181]
Greenhouse Gases Observing Satellite / Thermal And Near-infrared Sensor for carbon Observations - Fourier Transform Spectrometer (GOSAT/ TANSO- Fourier Transform Spectrometer (FTS) Satellite)	Operational	JAXA, Japan	10.0 × 10.0	3 days	758–775 1560–1720 1920–2080 5550–14300	Global	0.025	Poor	[32,33,41,57,145,146,147,148]
Global Ozone Monitoring Experiment–2 (GOME-2) / Metop satellites	Operational	EUMETSATEurope	40.0 × 40.0 80.0 × 40.0	29 days	240–790	Global	0.5	Very Good	[26,34,149,150,151,156,157,158,159,160,163,171,174]
SCanning Imaging Absorption SpectroMeter for Atmospheric CHartographY (SCIAMACHY) on board Envisat satellite	Non-operational	ESA, Europe	25.0 × 0.6 at the sub-satellite point (nadir) and 103.0 × 2.6 at the earth’s horizon (limb)	Full global coverage in 6 days	240–2400	Global (Nadir and limb view)	0.5	Very Good	[151]
Orbiting Carbon Observatory 2/OCO-2	Operational	NASA, USA	1.3 × 2.3	16 days	757–775	Global	0.04	Good	[165,166,167,169,170,172,173,174]
TROPOspheric Monitoring Instrument (TROPOMI)/Sentinel- 5p	Operational	ESA, Europe	7.0 × 7.0	16 days	270–500 675–775 2305–2385	Global	0.5	Very Good	[177,178]
Atmospheric Carbon-dioxide Grating Spectroradiometer (TanSat/ACGS)	Operational	Chinese space Agency, China	2.0 × 2.0	16 days	758–778 1594–1624 2042–2082	Global	0.04	Good	[180]
Hyperion imaging spectrometer/Earth Observing-1 (EO-1) satellite	Operational	NASA, USA	0.03	16 days	356–1058 852–2577	Global	10.72	Poor	[182,183]
Fluorescence Explorer/Fluorescence Imaging Spectrometer (FLEX/FLORIS)	Planned launch 2022	ESA, Europe	0.3 × 0.3	27 days	500–880	56°S – 75°N	0.3–0.2	Very Good	[22]
Geostationary Carbon Cycle Observatory (GeoCarb)	Planned launch 2022	NASA, USA	3.0 × 3.0	8 h	757–772 1591–1621 2045–2085 2300–2082	North and South America	0.05	Good	*-*
Meteosat Third Generation Sounder (MTG-S/Sentinel- 4)	Planned launch 2023	ESA, Europe	8.0 × 8.0	1 h	290–500 750–775	Europe	0.12	Good	*-*

## Supplementary Materials

The following are available online at https://www.mdpi.com/1424-8220/20/4/1144/s1, Table S1: Ground-based SIF observations, Table S2: Airborne SIF related studies published till 2019, Table S3: UAV based SIF Studies, Table S4: Spaceborne SIF studies.
