# Supplementary material for: Review of Top-of-Canopy Sun-Induced Fluorescence (SIF) Studies from Ground, UAV, Airborne to Spaceborne Observations
Source: Sensors (Basel). 2020 Feb 19;20(4):1144. doi: 10.3390/s20041144 (PMC7070282; doi:10.3390/s20041144)
Supplement: Supplementary file 1 [file sensors-20-01144-s001.pdf]

sensors

MDPI

Supply Material

Review of Top-of-Canopy Sun-Induced Fluorescence (SIF) Studies From Ground, UAV, Airborne to Spaceborne Observations

Subhajit Bandopadhyay *, Anshu Rastogi and Radosław Juszczak *

Laboratory of Bioclimatology, Department of Ecology and Environmental Protection, Faculty of Environmental Engineering and Spatial Management, Poznan University of Life Sciences, Poznan 60-649, Poland;

* Correspondence: subhajit.bandopadhyay@mail.up.poznan.pl (S.B.); radoslaw.juszczak@up.poznan.pl (R.J.)

Received: 18 January 2020; Accepted: 14 February 2020; Published: date

Table S1. Ground-based SIF observations.

Continent	Country	Reference	Fluorescence Retrieval Method	Device used	Target/Ecosystem	Aim of the study
**Detection of stress symptoms through SIF**						
North America	USA	[75]	FLD	Ground based FLD discriminator unit	Mature lemon trees	Plant water stress and fluorescence relationship
North America	USA	[76]	FLD	Fraunhofer Line Radiometer (FLR) prototype instrument	Control and DCMU treated palm trees and grape leaves	Fluorescence signal observation over control and DCMU treated platform
North America	USA	[80]	FLD	Plant Fluorescence Sensor (PFS)	Greenhouse-grown bean plants (*Phaseolus vulgaris* L.)	Relationship between degree of N_2 treatments with red and far red SIF
Europe	Spain	[78,79]	FLD	Prototype instrument (unnamed)	Maize plants	Understanding the physiological modifications due to DCMU and water stress
North America	USA	[81]	FLD	Plant Fluorescence Sensor (PFS)	Laurel oak leaves (*Quercus hamispherica*)	Herbicide induced stress experiment
North America	USA	[82]	FLD	Plant Fluorescence Sensor (PFS)	Corn and soybean plants	Herbicide induced stress experiment
Europe	Italy	[83]	SFM	HR2000 spectrometer, Ocean Optics, Dunedin, FL, USA	Bean (*Phaseolus vulgaris*) plants	Implementation of SFM method to derive the linear model for both fluorescence and reflectance over control and DCMU plots
Europe	Italy	[51]	FLD	HR2000 spectrometer, Ocean Optics, Dunedin, FL, USA	Bean (*Phaseolus vulgaris*) plants	Comparison of fluorescence signal over DCMU and control plots
Europe	Italy	[84]	FLD	HR2000 spectrometer, Ocean Optics, Dunedin, FL, USA	Poplar clone (*Populus deltoides* × *P. maximowiczii*)	Ozone stress detection experiment from leaf to canopy level using

						fluorescence signals
Europe	Italy	[85]	FLD	HR4000 spectrometers, Ocean Optics, Dunedin, FL, USA	White clover plants (*Trifolium repens* L. cv. Regal)	Ozone stress detection experiment from leaf to canopy level using fluorescence signals
**SIF theoretical observation**						
Australia	Australia	[48]	FLD	Multi-channel spectrometer MCS501, ZEISS International, Germany	Dock leaves (*Rumex obtusifolius* L.)	Examination of Kautsky fluorescence induction under natural and dark adopted conditions
**Comparison of active and passive methods for chlorophyll fluorescence measurements**						
North America	USA	[66]	FLD	ASD-FR FieldSpec Pro spectroradiometer, Analytical Spectral Devices, USA	Corn	Relation between passive reflectance and actively induced fluorescence
Europe	Germany	[65]	FLD principle and short-pass filter method	For active measurement: LI-6400-40 Li-COR Biosciences, USA leaf chamber fluorometer; For passive measurement: GER-1500, USA field portable spectroradiometer	Wheat	Comparison between active fluorescence and passive method based SIF measurements over wheat from leaf to canopy scale
**SIF dynamics in relation to PAR conditions**						
Europe	France	[72]	FLD	Prototype instrument (unnamed)	Single bean leaf	Relationship between fluorescence yield and reflectance in changing PAR conditions
Europe	France	[73]	FLD	Passive Multi-wavelength Fluorescence Detector (PMFD)	Scots pine trees in the boreal forest	Relation between dynamic PAR and SIF signals
Asia	China	[74]	FLD	ASD-FR FieldSpec Pro spectroradiometer, Analytical Spectral Devices, USA	Wheat (*Triticum aestivum* L.) and Japan Creeper (*Parthenocissus tricuspidata*)	Relation between dynamic PAR conditions with SIF at 688 nm and SIF at 760 nm
**Development of SIF retrieval methods and SIF simulations through models**						
Europe	Spain	[54]	iFLD	ASD-FR FieldSpec Pro spectroradiometer, Analytical Spectral Devices, USA	Hibiscus leaf	Development of iFLD method
Europe	Italy	[69]	Quadratic functions	Double monochromator	Plant leaves	O_2B fluorescence signal retrieval
Europe	Germany	[68]	SVD	HR4000 spectrometer, Ocean Optics, Dunedin, FL, USA	Paddy field (*Oryza sativa* L. var. japonica)	Asses the capacity of SVD statistical method to retrieve O_2A SIF signal

Europe	The Netherlands	[71]	SCOPE Model	HR4000 spectrometer, Ocean Optics, Dunedin, FL, USA	Rice (*Oryza sativa* L.) field, alfalfa (*Medicago sativa* L.) field. Two plots of lawn grass: a mixture consisting of *Festuca rubra, Lolium perenne* and *Poa pratensis*	Comparison of Modelled and measured SIF estimations
Asia	China	[47]	SCOPE Model	N/A	N/A	Understanding the impact of reflectance and SNR on SIF retrievals
Europe	Italy	[67]	eFLD	Double monochromator	Single leaves of *Lycopersicon esculentum, Cucurbita pepo, Cucumis sativus,* and *Epipremnum aurea*	First preliminary experiment on eFLD method
**SIF time series for different canopies**						
Europe	France	[87]	Radiance based filling-in method	TriFLEX	Sorghum field	Observation of fluorescence, NDVI, and PRI characters during entire growing period
Europe	Italy	[88]	SFM	HR4000 spectrometer, Ocean Optics, Dunedin, FL USA	Sugar beet, grassland and lawn carpet canopies	Time series fluorescence measurement during the growing cycle
Europe	Italy	[89]	SFM	Three portable field spectrometers (two HR4000 and one QE65000, Ocean Optics, Dunedin, FL, USA	Cropland, grassland, needleleaf forest, deciduous broadleaf forest	Understand the variations of magnitude in emitted fluorescence signals in both red and far-red regions
**SIF used for net CO_2 and GPP fluxes estimation of different canopies**						
North America	USA	[91]	FLD	Stationary fluorescence Detector similar to PFS	Leaves of three species (i.e., *Liquidambar styracifiua* L., *Pinus taeda* L., *Zea mays* L.)	Fluorescence and net CO_2 assimilation rate relationship
Europe	Spain	[29]	FLD	Prototype instrument (unnamed)	Single bean leaf, natural grassland, maize (*Zea mays* L.) cropland	Relationship between SIF and gas exchange from leaf to canopy level
Europe	Switzerland	[92]	FLD	ASD FieldSpec Pro spectroradiometer, Analytical Spectral Devices, USA	Corn (*Zea mays* L.), winter wheat (*Triticum vulgare*) and beans (*Phaseilus vulgaris*)	Estimation of GPP using SIF signal
North America	USA	[93]	SFM	FluoSpec2	Temperate deciduous forest	Representing SIF-GPP relationship at diurnal and seasonal scales

Europe	Germany	[58]	In-filling method	HR4000 spectrometer, Ocean Optics, Dunedin, FL USA	Mediterranean savanna ecosystem, with low density of oak trees (mostly *Quercus ilex*)	Improved estimation of GPP from SIF and PRI
Europe	Germany	[94]	SCOPE Model	HR4000 spectrometer, Ocean Optics, Dunedin, FL USA	Mediterranean grassland	SIF-GPP relationship under nitrogen–phosphorous (NP) treatment conditions
Europe	Germany	[95]	SCOPE Model	HR4000 spectrometer, Ocean Optics, Dunedin, FL USA	Mediterranean grassland	SIF-GPP relationship under nitrogen–phosphorous (NP) treatment conditions
Europe	Austria	[96]	SCOPE Model	S-FLUO Box (FZJ Jülich, Germany)	Mediterranean pine forest	SIF-GPP relationship under short-term intense heat wave condition
**SIF measuring systems (description and comparison)**						
Europe	Italy	[24]	3FLD for O_2A sFLD for O_2B	1. Multiplexer Radiometer Irradiometer (MRI) (Milan, Italy) 2. S-FLUO Box (FZJ Jülich, Germany) 3. FUSION (Greenbelt, MA, USA) 4. TriFLEX (Paris, France)	Lawn grass, (*Festuca arundinacea*)	Comparative analysis among four ground based spectroradiometers to estimate SIF in support of ESA FLEX mission
North America	USA	[90]	SFM and SVD	FluoSpec2	cropland and forest	Capacity of FluoSpec2 to measure SIF signals over different ecosystems

Table S2. Airborne SIF related studies published till 2019.

Continent	Country	Reference	Fluorescence Retrieval Method	Device type	Target/Ecosystem	Aim of the study
**FLI -** Fluorescence Line Imager						
North America	Canada	[100]	Fluorescence Line Height (FLH)	Multispectral Fluorescence Line Imager	Marine phytoplankton	Mapping of phytoplankton biomass
**ROSIS -** Reflective Optics System Imaging Spectrometer						
Australia	Australia	[48]	FLD	Multispectral Imaging spectrometer	Winter cereal, corn, barley cultivated lands at Barrax site	Understand and analyze the spatial pattern of SIF
**CASI -** Compact Airborne Spectrographic Imager						
North America	Canada	[59]	FRT	Hyperspectral imaging spectrometer	Sugar maple forest	Understanding the relationship between airborne hyperspectral canopy reflectance spectra and ground reflectance spectra using Fluorescence-Reflectance-Transmittance model
North America	Canada	[102]	FRT		Sugar maple forest	Measurements of spectral reflectance under artificial and natural light conditions to demonstrate the effects of natural chlorophyll fluorescence
Europe	Spain	[103]	FLD		Maize (*Zea mays* L.)	Variability of SIF during different nitrogen induced stressed conditions
Europe	Italy	[104]	Modified FLD with absorption at 762 nm		Maize (*Zea mays* L.)	Understanding the relationship between SIF and water stress
**AISA** - Airborne Imaging Spectrometer for Applications						
North America	USA	[66]	FLD	Hyperspectral imaging spectrometer	Maize (*Zea mays* L.)	Relationship between SIF and reflectance under different nitrogen conditions
North America	USA	[106]	FLD		Maize (*Zea mays* L.)	Comparison between ground based and modelled SIF values under different nitrogen conditions
Europe	Italy	[30]	FLD		Maize (*Zea mays* L.) and Sorghum (*Sorghum bicolor* L.)	Water stress detection through SIF signal
**APEX -** Airborne Prism Experiment						
Europe	Switzerland	[112]	FLD	Hyperspectral imaging spectrometer	two tree species, sugar beet and winter wheat	The impact of varying irradiance on estimating of vegetation indices (i.e., NDVI, PRI) and SIF signal
Europe	Switzerland	[113]	SCOPE model		perennial grassland, cropland and mixed temperate forest	To understand the SIF_{760} and GPP relationship at leaf to canopy scale

**AirFLEX** fluorescence sensor						
Europe	France	[50]	cFLD	Multispectral Imaging spectrometer	Cropland (alfalfa, sugar-beet, wheat)	Sensitivity analysis to evaluate the effects of different flight altitudes (from 300 to 3000 m above ground level) in SIF band depth
Europe	France	[108]	FLD		Various crops	Development of a correction model of altitude effects on the depth on oxygen absorption bands
**HyPlant**						
Europe	Germany	[14]	3FLD	Hyperspectral imaging dual spectrometer system	Forest, Grassland, Croplands (sugar beet, maize, potato)	To capture the large spatial variability of different vegetation types from SIF signals
Europe	Italy	[35]	SVD and a physically based approach that incorporates explicit atmospheric RTM using MODTRAN5		Commercial grass carpets (control and herbicide induced)	Linking the SIF signals at red and far-red region to the variations in actual photosynthetic efficiency
Europe	Germany	[116]	iFLD		Cropland (sugar beet, maize, rape seed, potato)	Improving the estimations of GPP using SIF signal compare to greenness indices
Asia	China	[117]	iFLD		Cropland (cotton and vegetables (i.e., sweet potato), Chinese cabbage, thyme, pumpkin)	Understanding SIF-GPP relationship over different species and different canopy structures
Europe	Italy	[118]	SVD		Managed loblolly pine (*Pinus taeda* L.) forest	Estimation of tree age based on red and far-red SIF signals
North America	USA	[120]	SVD		Managed loblolly pine (*Pinus taeda* L.) forest	SIF signals to understand the plant physiological process and forest ecosystem health.
Europe	Poland	[122]	SFM		Grassland, forest and peatland ecosystems and peatland plant communities	To understand SIF vs. vegetation indices relationship at ecosystem level and plant community level
Europe	Luxembourg	[121]	FLD		Commercial grass treated with VaporGard and kaolin antitranspirants	Investigation of water stress using SIF and TIR images over a day
Europe	Italy	[119]	SFM		Mid-latitude plain mixed forest	To understand the relationship between GPP and APAR with both SIF bands
**CFIS** - Chlorophyll Fluorescence Imaging Spectrometer						
North America	USA	[126]	FLD	Hyperspectral imaging spectrometer	Croplands (maize, sorghum, winter wheat, rye, oats) and different land covers like forest, grass seed, peas, barre, water, wetlands etc.	Demonstrating the technical aspects of CFIS airborne imaging spectrometer for the validation of OCO-2 satellite

**Micro-hyperspectral Imaging Sensor**						
North America	Canada	[123]	3FLD	Micro-hyperspectral imaging sensor	Citrus crop (*Citrus sinensis* L. cv. Powell)	Understanding seasonal stability of SIF as an indicator of net photosynthesis in the context of precision agriculture
Europe	Spain	[124]	FLD		Almond orchard	Impact of structural heterogeneity on SIF and on water stress index
Europe	Spain	[125]	FLD		Cropland (wheat)	Impact of nitrogen concentration under irrigated and rain fed Mediterranean conditions
**Non-imaging spectroradiometer system**						
Europe	Switzerland	[110]	FLD	Non-imaging spectroradiometer (ASD FieldSpec HR) Analytical Spectral Devices, USA	Cropland (sugar beet)	Impact of atmospheric effects on SIF retrievals.
Europe	Germany	[111]	3FLD		Cropland (winter wheat and sugar beet)	Improving the estimation of GPP from SIF and PRI using Monteith's light use efficiency (LUE) concept

Table S3. UAV based SIF Studies**.**

Continent	Country	Reference	Fluorescence Retrieval Method	Sensor	Target/ Ecosystem	Aim of the study
Europe	Spain	[129]	In-filling method	Multi-spectral camera (MCA-6, Tetracam, USA)	olive, peach, and orange orchards	To detect the water deficiency
Europe	Spain	[31]	3FLD	Micro-Hyperspectral imager (Micro-Hyperspec VNIR model, Headwall Photonics, MA, USA)	Orchard tree	Sensitivity analysis of water stress level and stomatal conductance through chlorophyll fluorescence
Europe	Spain	[130]	3FLD	Micro-Hyperspectral imager (Micro-Hyperspec VNIR model, Headwall Photonics, MA, USA)	Non-irrigated vineyards	Understanding the relationship between steady-state fluorescence and net photosynthesis from leaf to canopy level
Europe	Spain	[131]	FLD	Micro-Hyperspectral imager (Micro-Hyperspec VNIR model, Headwall Photonics, MA, USA)	Tree canopies of citrus orchards	Understanding the impact of spatial resolution on chlorophyll fluorescence retrieval from heterogeneous canopies
Europe	Spain	[132]	FLD	Micro-Hyperspectral imager (Micro-Hyperspec VNIR model, Headwall Photonics, MA, USA)	Olive plants	Detection of disease infection using fluorescence, temperature and narrow-band spectral indices
Europe	Italy	[127]	3FLD	small hyperspectral imager (HyUAS)	Mixed forest, croplands, meadows and over some non-fluorescent targets like soil	Discussed the technical aspects as well as for the purpose of calibration and validation of present and upcoming spaceborne and airborne missions by ESA
Europe	UK	[128]	Fv/Fm ratio	Piccolo Doppio UAV system	Mature oak forest	Free Air CO_2 Enrichment (FACE) experiment thorough SIF signal

Table S4. Spaceborne SIF studies.

Continent	Country	Reference	Fluorescence retrieval method	Satellite Name	Target/Ecosystem	Aim of the study
			**MERIS and/or MODIS**			
North America	Canada	[136]	Fluorescence Line Height (FLH)	MERIS	Phytoplankton	Detection and mapping of phytoplankton from fluorescence signal
North America	Canada	[137]	Fluorescence Line Height (FLH)	MODIS	Phytoplankton	Modelling of fluorescence from MODIS data
North America	Canada	[135]	Fluorescence Line Height (FLH)	MERIS and MODIS	Bright plankton blooms	Comparison of MERIS and MODIS data potential to estimate fluorescence
Europe	Spain	[140]	cFLD	MERIS	Barrax ecosystem	Estimation of SIF from MERIS data using new retrieval algorithm
			**GOSAT**			
North America	USA	[41]	Filling-in of the potassium (K) I solar Fraunhofer line method near 770 nm	GOSAT	Global vegetation	Mapping of SIF in a full seasonal cycle for several different locations of the globe
North America	USA	[32]	Through radiance spectra measurement in the red spectral range	GOSAT	Boreal forests, savannas, croplands, high-latitude needleleaf forests.	To understand the uncertainties and additional dependencies such as climatic factors in global GPP estimation from SIF
Europe	Germany	[33]	SVD	GOSAT	Global vegetation	Development of new methodology to retrieve SIF through the modeling of the in-filling of FLD lines
Asia	Japan	[144]	Filling-in method	GOSAT and OCO-2	Global ecosystems including non-vegetative areas	Identifying the criteria for selecting vegetation-free areas to evaluate the zero-level offset comparing GOSAT-FTS and OCO-2
North America	USA	[145]	FLD	GOSAT	Amazonian forest	Impact of water stress on plant productivity using SIF and GPP
North America	USA	[148]	FLD	GOSAT	Southern Amazonia	Understanding the seasonal changes in carbon balance from SIF data
North America	USA	[146]	SCOPE Model	GOSAT	Global vegetation	Simulation of SIF through Community Land Model version 4 for evaluating photosynthesis
North America	USA	[147]	Average of two bands (757 nm and 771 nm) and two polarizations (p and s) techniques	GOSAT	Global ecosystems (i.e., tropical forests within the Amazon Basin, northern croplands and deciduous forests)	Estimation of global GPP over different ecosystems from global SIF data
Europe	Germany	[57]	GARLiC	GOSAT	Global vegetation	Development of new SIF retrieval method from GOSAT data
			**GOME-2**			
North America	USA	[34]	Fitting window algorithm	GOME-2	Global vegetation	Development of new SIF retrieval technique to retrieve global far-red fluorescence
Europe	Germany	[149]	Fitting window algorithm and SCOPE for SIF modelling	GOME-2	USA Corn belt	Estimation of vegetation photosynthetic process, particularly carboxylation process using SIF data
North America	USA	[150]	Fitting window algorithm	GOME-2	Different global ecosystems (i.e., savannas, evergreen broadleaf, croplands, mixed forests)	To track the seasonal cycle of photosynthesis (in terms of GPP) and modeling of carbon uptake
Europe	Germany	[26]	Spectral radiance measurements	GOME-2	USA croplands and European grasslands	To show that chlorophyll fluorescence would be a unique benchmark to improve our global models for agricultural productivity and climate impact on crop yields

North America	Canada	[153]	Fitting window algorithm	GOME-2	Global vegetation	Understanding the relationship between angular normalized SIF values with GPP under sun and shaded conditions
Europe	Germany	[151]	Linear method	GOME-2 and SCIAMACHY	Global vegetation	Development of new SIF retrieval method from GOME-2 and SCIAMACHY data
North America	USA	[152]	SFLs method	GOME-2 and SCIAMACHY	Global vegetation	Development of new SIF retrieval method applied over GOME-2 and SCIAMACHY data
North America	USA	[154]	Fitting window algorithm	GOME-2	Maize cropland	Investigation of the potential of space borne SIF data to describe crop phenology and evaluated three GPP modeling approaches
Asia	China	[155]	Fitting window algorithm	GOME-2	Harvard forest	Tracking of SIF-GPP relationship from leaf to ecosystem level during seasonal variation
North America	USA	[156]	Fitting window algorithm	GOME-2	USA Croplands	Monitoring crop yield and crop productivity using SIF
North America	USA	[160]	Fitting window algorithm	GOME-2	Croplands, grasslands, mixed forest	Understanding the relationship between SIF, NDVI and FAPAR, GPP during drought period
North America	USA	[161]	Fitting window algorithm	GOME-2	Great Plains	Monitoring the drought dynamics from SIF anomalies
Asis	China	[162]	Fitting window algorithm	GOME-2	Great Plains	To understand the sensitivity of SIF during short term and long term drought conditions
Europe	Germany	[158]	FLD	GOME-2	Boreal forests of mid to high latitude	Understanding the seasonal photosynthetic dynamics using SIF
Asia	China	[159]	GARLiC	GOME-2	Cropland, grassland, evergreen needle forest, deciduous broadleaf forest, and woody shrublands	Relationship between SIF-GPP in short-term seasonal scale over different biomes of the world
North America	USA	[164]	PCA with simplified RTM model and FLD	GOME-2 and GOSAT	Northern high latitude forests	Understanding the capacity of SIF data to capture the hysteresis and plant phenology during the seasonal cycle
Asia	India	[157]	Raw data at 740 nm wavelength	GOME-2	Indo-Gangetic plain	Investigating the net primary productivity of crops using SIF data
Asia	China	[163]	Linear Method	GOME-2 and GOSAT	Indo-Gangetic wheat field	Impact on wheat production due to heat stress
North America	USA	[181]	Machine learning approach	GOME-2 and MODIS	Global vegetation	Reconstruction of SIF data from surface reflectance
				**OCO-2**		
North America	USA	[169]	SVD	OCO-2	Global vegetation	Understanding the potential of OCO-2 data to retrieve SIF
Europe	Ireland	[171]	FLD	OCO-2 and GOME-2	Tundra region	To track the photosynthetic activity from SIF and EVI
North America	USA	[172]	FLD	OCO-2	Savanna grassland	Effect of environmental conditions on spaceborne SIF and tower based GPP
North America	USA	[166]	FLD	OCO-2	Temperate forest	Demonstrating the ability of OCO-2 SIF data to estimate GPP
North America	USA	[170]	FLD	OCO-2	Evergreen needleleaf forests, evergreen broadleaf forests, shrublands, and savannas	Investigation of biome specific SIF-GPP relationship using OCO-2 SIF data
Asia	China	[173]	FLD	OCO-2	Mixed forest, woody savannas, evergreen needleleaf forest	Effect of BRDF on SIF-GPP relationship

North America	USA	[174]	Fitting window algorithm	OCO-2 and GOME-2	Shrubland, savanna and woody savanna, mixed forest, evergreen needleleaf forest and grasslands	Tracking of seasonal and inter-annual dynamics of GPP from SIF in a dryland ecosystem
Europe	France	[175]	PCA	OCO-2 and GOME-2	Global vegetation	Understanding the impacts of acquisition characteristics and processing chain to estimate GPP model optimization process
North America	USA	[176]	Data driven Cubist regression tree model	OCO-2 and MODIS	Global vegetation	Development of new SIF products (GOSIF) high spatial and temporal resolutions
			**TROPOMI**			
Europe	Germany	[177]	Filling-in method	TROPOMI	Global vegetation	Estimation of uncertainty in SIF retrieval using TOPOMI in respect to current in-flight spectrometer such as GOME-2
North America	USA	[178]	Filling-in method	TROPOMI	Global vegetation	Inter-sensor comparison between TROPOMI and OCO-2
North America	USA	[179]	Backward eliminating principal component method	TROPOMI	Tropical Amazonian forest	Investigation of plant photosynthesis process in terms of SIF during dry season
			**TanSat**			
Asia	China	[180]	SVD	TanSat	Global vegetation	Understanding spatio-temporal pattern and relationship between SIF and NDVI, EVI and GPP
			**Hyperion**			
Asia	China	[183]	FLD	Hyperion	Forest	Understanding the relationship between SIF and NDVI
Asia	India	[182]	New method that disentangles the signals between vegetated and non-vegetated areas (ex. urban, waterbody) from radiation ratio method	Hyperion	Vegetative areas of Kolkata city, India	Proposing a methodology for the estimation of chlorophyll fluorescence from hyperspectral images
